# Kounis Syndrome in the Modern Era: A Comprehensive Review of Allergic Acute Coronary Syndromes

**DOI:** 10.3390/jcm15166417

**Published:** 2026-08-19

**Authors:** Lucio Giuseppe Granata, Giuseppe Andò, Marcello Marchetta, Simona Giubilato, Nicholas G. Kounis, Cesare de Gregorio

**Affiliations:** 1 Cardiology Division, Garibaldi-Nesima Hospital, Azienda Ospedaliera di Rilievo Nazionale e di Alta Specializzazione (ARNAS) Garibaldi, 95122 Catania, Italy; 2 Department of Cardiology, Azienda Ospedaliera Papardo, 98158 Messina, Italy; 3 Department of Clinical and Experimental Medicine, University of Messina, 98125 Messina, Italy; 4 Department of Cardiology, Policlinico Tor Vergata, Viale Oxford, 81, 00133 Rome, Italy; marcello.marchetta1997@gmail.com; 5 Cardiology Division, Cannizzaro Hospital, 95126 Catania, Italy; 6 Department of Cardiology, Faculty of Medicine, University of Patras, 26500 Patras, Greece; ngkounis@otenet.gr; 7 Cardiology Unit, Department of Clinical and Experimental Medicine, Heart Failure and Cardiomyopathy Outpatient Office, G. Martino Hospital Messina, 98125 Messina, Italy

**Keywords:** Kounis syndrome, allergic myocardial infarction, acute coronary syndrome, MINOCA, coronary vasospasm

## Abstract

Kounis syndrome (KS) is a largely underdiagnosed cause of acute coronary syndromes triggered by allergic or hypersensitivity reactions. The syndrome results from complex immune cell activation with the release of vasoactive and prothrombotic mediators leading to coronary vasospasm (type I KS) or thrombosis of plaque (type II KS), stent (type III KS) or coronary artery bypass graft (type IV KS). Despite increasing recognition over the past few decades, its pathophysiological mechanisms, diagnostic boundaries, and therapeutic implications remain incompletely understood. A comprehensive diagnostic approach, including signs, symptoms, biochemical findings, electrocardiography, echocardiography, coronary angiography, and multimodality imaging, as well as invasive assessment in selected cases, can be recommended, although its implementation in routine practice remains limited. Available data indicate that angiographically documented epicardial coronary spasm is observed in only a minority of patients, while normal or non-obstructive coronary arteries are frequently encountered. Emerging data from provocative testing, invasive coronary functional assessment, cardiac magnetic resonance and nuclear imaging suggests that coronary microvascular dysfunction may contribute substantially to the clinical phenotype, expanding the traditional concept of allergic epicardial vasospasm. Current evidence supports the recognition of type I KS as a distinct allergic vasomotor acute coronary syndrome within the myocardial infarction non-obstructive coronary artery (MINOCA) spectrum, deserving greater recognition in future diagnostic classifications and clinical practice guidelines. This narrative review critically appraises current evidence, integrating historical perspectives with contemporary insights into classification, pathophysiology, triggers, diagnostic strategies and therapeutic approaches, focusing on the most frequent manifestation represented by the vasospastic variant.

## 1. General Overview: Epidemiology, Classification and Outcomes

### 1.1. Definition and Historical Background

Myocardial ischemia or infarction occurring in a close temporal and causal relationship with an allergic, anaphylactic, or anaphylactoid reaction is defined as allergic myocardial infarction (MI) [1].

For many years allergic phenomena were not recognized as a potential cause of MI. The first case was reported in 1938 following the administration of antitetanus serum [2], and the few subsequent reports over the following years described similar events almost exclusively associated with this agent [3,4,5,6]. In 1950, Pfister reported the first case of penicillin-induced MI in a 49-year-old man who developed an extensive anteroseptal MI during a prolonged urticarial reaction that followed penicillin administration [7]. Several decades later, Constantinides hypothesized that allergic reactions might promote coronary plaque rupture based on the observation that circulating mast cells could infiltrate disrupted endothelial junctions within atherosclerotic plaque [8,9]. Subsequent studies demonstrated the presence of mast cells within culprit coronary lesions, particularly in plaques complicated by erosion or rupture, supporting a mechanistic link between allergic inflammation and acute coronary events [10].

Allergic angina and MI were first systematically described in 1991 by Nicholas G. Kounis and George M. Zavras, who reported the association of chest pain and allergic reactions accompanied by clinical and laboratory evidence of myocardial ischemia [11].

Since then, this entity has become widely known as Kounis syndrome (KS), defined as an acute coronary syndrome (ACS) triggered by an allergic reaction and characterized by the coexistence of allergic manifestations and myocardial ischemia with electrocardiographic abnormalities and normal or elevated cardiac troponin levels. In 1998, Braunwald incorporated allergic angina into the spectrum of dynamic coronary obstruction, recognizing that mediators released during allergic reactions, including histamine and leukotrienes, may induce coronary vasospasm [12]. Allergic reactions are now recognized as well-established triggers of ACS [1]. However, KS is not a single-organ vascular disorder but a multisystem and multidisciplinary disease. Today, it is characterized as a distinct kind of acute vascular syndrome that affects not only the coronary arteries but also the cerebral, mesenteric, peripheral and venous systems [13].

### 1.2. Epidemiology

KS is widely underdiagnosed and underreported because of limited awareness and insufficient clinical recognition. The absence of national and international registries, prospective studies, and dedicated guidelines contributes to the paucity of robust epidemiological data and standardized diagnostic and therapeutic pathways. The syndrome has been reported across all ethnic groups and geographic regions. Southern Europe (including Italy, Greece, Turkey, Spain) and Japan appear to be more frequently represented, possibly reflecting greater diagnostic awareness, but also environmental factors, inappropriate drug use, or suboptimal preventive measures [1,14]. A potential genetic contribution has been suggested, linking KS-related vasospasm to heterozygous variants of the E148Q gene variant, supporting the hypothesis that dysregulated innate immunity may contribute to disease expression, although current evidence remains anecdotal and largely hypothesis-generating [15].

In the first prospective study on KS, conducted in Turkey among patients presenting to the emergency department, the incidence of the syndrome was estimated at 19.4 cases per 100,000 admissions (27/138,911) and accounted for 3.4% (27/793) of patients presenting with allergic reactions [16]. In the Achaia region of Greece, with a population of approximately 300,000, 52 cases of KS were reported over four years, corresponding to an annual incidence of 4.33 per 100,000 inhabitants [17]. During a three-year period (2006–2009), among 3876 patients with suspected myocardial infarction undergoing coronary angiography in a tertiary hospital in Istanbul, eight cases of KS were identified, corresponding to a prevalence of approximately 0.2% [18]. The largest epidemiological study to date was conducted in the United States using the National Inpatient Sample (NIS), which includes a nationally representative stratified sample of hospital discharges. Among 235,420 hospitalizations for anaphylactic or hypersensitivity reactions between 2007 and 2014, KS was identified in 1.1% of cases (2616/235,420), patients with KS had a significantly higher in-hospital mortality compared to those without KS (7% vs. 0.4%), were typically older, more often male and White, and experienced longer hospital stays. They also showed higher rates of cerebrovascular events, arrhythmias (30% vs. 12.4%), deep vein thrombosis, and a greater need for coronary angiography, percutaneous coronary intervention (PCI), or coronary artery bypass grafting (CABG) [19].

A retrospective Italian study including 24,535 patients presenting with allergic reactions between 2008 and 2020 identified anaphylaxis in 444 patients, of whom 2% (9/444) were diagnosed with KS [20]. In a cohort of 300 patients presenting with anaphylaxis evaluated with echocardiography in the emergency department, elevated serum troponin-I identified myocardial injury in 7.3% of cases, attributable to various etiologies including KS, Takotsubo syndrome, and other cardiomyopathies [21].

More recently, Anastasopoulou et al. conducted a prospective study including 200 patients presenting with type I hypersensitivity reactions between 2018 and 2021 at the Emergency Department of the University Hospital of Patras (Greece) [22]. Applying rigorous inclusion and exclusion criteria, myocardial injury was identified in 12% of patients, with half of these cases (6%, 12/200) attributable to KS. These findings underscore the importance of actively screening for KS during allergic reactions, even mild ones, using electrocardiography, echocardiography, high-sensitivity troponin, serum tryptase, and coronary angiography when clinically indicated [23]. It is also important to consider the intra-hospital setting, as allergic reactions account for approximately 3–4% of coronary spasms occurring in the perioperative period [24].

KS can affect all age groups, ranging from children to the elderly, with reported ages spanning from 2 to 90 years [1,10,25,26,27,28], with a mean reported age of approximately 54 years, and a predominance in males (64–74.3%) [29,30,31,32,33,34].

### 1.3. Contemporary Classification

All forms of KS are triggered by allergic or hypersensitivity reactions but differ in their downstream effects on the coronary circulation. Biochemical evidence of myocardial injury occurs in approximately 57–73% [33,35,36]; elevation of high-sensitivity cardiac troponin (hs-cTn) and/or creatine kinase-MB (CK-MB) reflects myocardial ischemia or infarction resulting from the severity and duration of coronary luminal narrowing [37].

Classically, three subtypes have been described, and a fourth subtype has recently been added [1,17,38,39]:

Type I KS: coronary vasospasm;

Type II KS: coronary atherothrombosis of a pre-existing vulnerable plaque;

Type III KS: coronary stent thrombosis or coronary stent restenosis;

Type IV KS: coronary artery bypass graft thrombosis or spasm.

**Figure 1:**
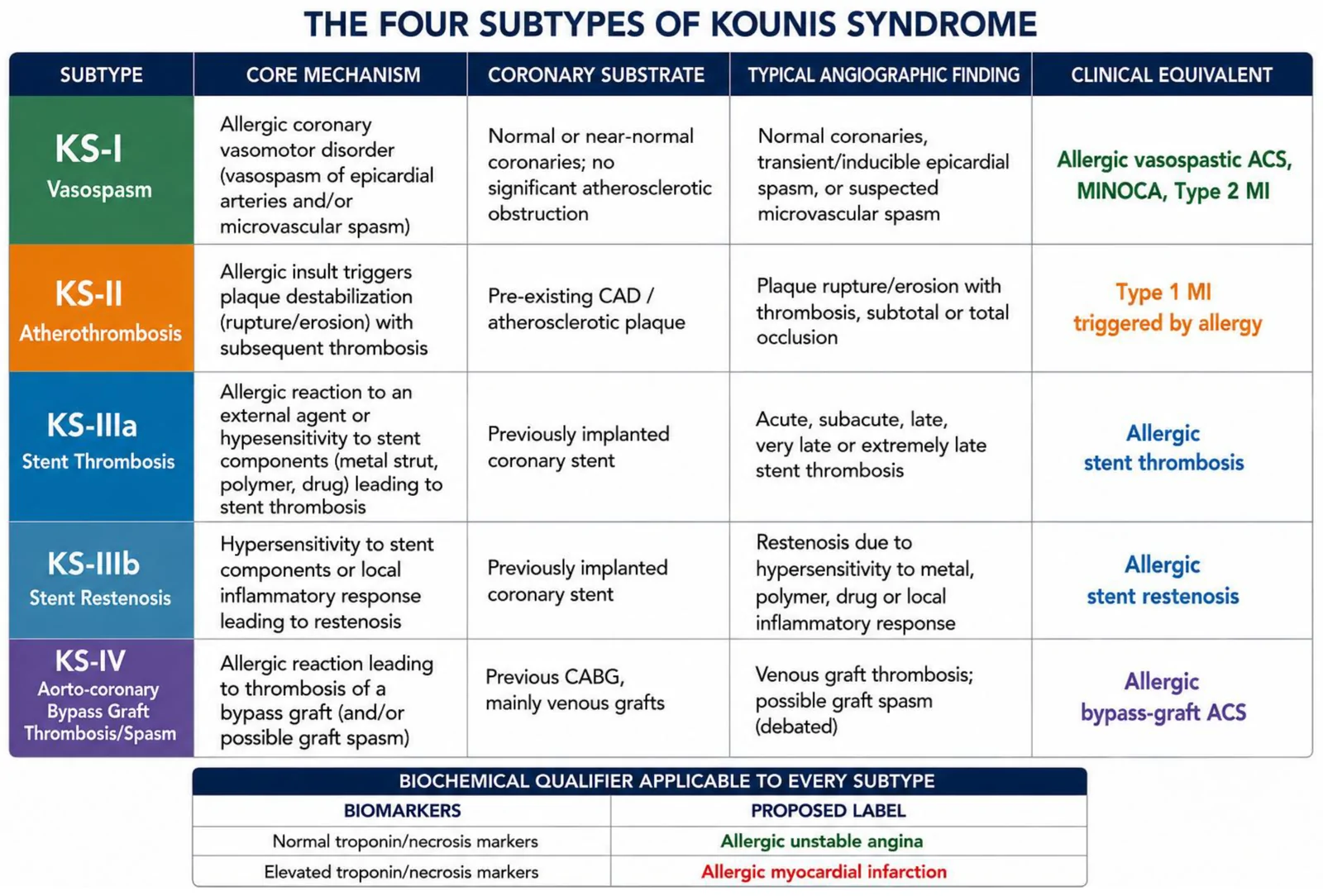
Contemporary pathophysiological and clinical classification of KS. The figure summarizes the four currently recognized subtypes of KS according to their underlying mechanisms, coronary substrates, angiographic characteristics, and clinical equivalents. Type I KS is characterized by allergic coronary vasomotor dysfunction involving epicardial and/or microvascular spasm in patients without significant obstructive coronary artery disease and corresponds to an allergic vasospastic ACS phenotype within the contemporary MINOCA spectrum. Type II KS occurs when an allergic reaction triggers destabilization of a pre-existing atherosclerotic plaque, leading to plaque rupture or erosion with thrombosis and type 1 myocardial infarction. Type III KS includes hypersensitivity-mediated stent failure and is subdivided into IIIa (allergic stent thrombosis) and IIIb (allergic stent restenosis). Type IV KS represents the bypass-graft counterpart and encompasses allergic thrombosis and/or vasospasm involving coronary artery bypass grafts. The lower panel illustrates a biochemical qualifier applicable to all KS subtypes, distinguishing allergic unstable angina from allergic myocardial infarction according to the absence or presence of myocardial necrosis biomarkers. Abbreviations: ACS, acute coronary syndrome; CABG, coronary artery bypass grafting; CAD, coronary artery disease; KS, Kounis syndrome; MI, myocardial infarction; MINOCA, myocardial infarction with non-obstructive coronary arteries.

Given the increasing recognition of distinct pathophysiological mechanisms underlying allergic ACS, Figure 1 shows contemporary classification of KS (Figure 1). This framework integrates the currently recognized subtypes with their corresponding coronary substrates and clinical phenotypes, aligning the traditional classification with current concepts of myocardial infarction with non-obstructive coronary arteries (MINOCA).

Of note, although KS is traditionally classified into distinct subtypes, overlap between phenotypes may occur in the same patient. Indeed, allergic coronary vasospasm, plaque destabilization, and thrombotic complications should be regarded as different manifestations of a common mast cell-driven pathophysiological continuum rather than mutually exclusive entities. Consequently, a patient initially presenting with type I KS may subsequently develop plaque rupture or coronary thrombosis, while vasospastic and thrombotic mechanisms may coexist during the same clinical event, generating hybrid phenotypes that do not fit neatly into the conventional classification [40].

### 1.4. Evidence from Published Cases and Clinical Cohorts

Over the past decade, the evidence base supporting KS has expanded substantially, progressing from isolated case reports to large systematic reviews, pooled analyses, and real-world clinical cohorts. Although type I KS remains the most frequently reported phenotype, recent studies have highlighted a progressive increase in the recognition of type III, likely reflecting greater awareness of allergic stent-related complications and wider adoption of intracoronary imaging techniques and thrombus histopathology [41]. Nevertheless, significant heterogeneity persists among published series regarding case ascertainment, subtype classification, diagnostic criteria, and outcome reporting. Consequently, the true epidemiological distribution of KS subtypes remains uncertain.

Table 1 summarizes the principal reviews and clinical series published to date, illustrating the evolution of reported subtype prevalence and mortality. Notably, while type I KS consistently represents the predominant phenotype, the highest mortality rates have been reported among patients with type III KS, underscoring the particularly severe clinical implications of allergic stent thrombosis.

### 1.5. Special Populations

Special populations may exhibit distinctive features of KS. In elderly patients, polypharmacy, atherosclerotic burden, endothelial dysfunction, and plaque vulnerability may favor type II and III presentations, whereas pediatric KS predominantly presents as type I vasospastic disease. Pregnancy and the peripartum period represent a unique immune and hemodynamic setting that may enhance coronary vasoreactivity, while patients with chronic kidney disease may be particularly vulnerable because of chronic inflammation, endothelial dysfunction, and repeated exposure to potential allergens. Other potentially susceptible groups include highly atopic, autoimmune, and oncological patients [43]. KS may also occur in transplant candidates, particularly in the perioperative setting, where exposure to multiple potential allergens, including anesthetic agents, antibiotics, and neuromuscular blocking agents, may complicate identification of the culprit trigger. Moreover, predominant cardiovascular manifestations with few or absent typical allergic signs may hinder recognition and lead to misclassification as a conventional perioperative acute coronary event [44,45].

### 1.6. Clinical Outcomes and Prognosis

Contemporary cohorts suggest that the majority of survivors experience complete clinical recovery, particularly in type I KS, where coronary vasomotor dysfunction is usually transient and reversible and the prognosis is generally excellent [36,42]. One of the most intriguing observations emerging from contemporary datasets is the apparent paradox between the generally favorable prognosis of KS and its unexpectedly high burden of life-threatening complications. Despite mortality rates remaining below 10% in most series, malignant ventricular arrhythmias and cardiac arrest are reported with unexpected frequency [32]. This observation is particularly striking given that a substantial proportion of patients exhibit angiographically normal or non-obstructive coronary arteries.

Early reviews reported major complications in approximately 8.5% of patients, whereas more recent systematic analyses suggest rates approaching 27%. Cardiogenic shock occurs in approximately 2.3–8.5% of cases, while respiratory failure is reported in about 5% [29,35]. Cardiac arrest is one of the most feared complications, occurring in 6.3–11.8% of patients [29,34]. Malignant arrhythmias include ventricular fibrillation (10.2%), pulseless electrical activity (2.1%), and asystole (1.3%), highlighting the potential for sudden cardiac death even in patients without obstructive coronary artery disease [32]. Cardio-respiratory arrest has been reported in approximately 6.7% of cases, whereas conduction disturbances and severe bradyarrhythmia may further contribute to hemodynamic instability [35].

Mortality rates range from 2.9% to 5.5%, although prognosis is generally favorable in most cases [29,30,31,32,33,34]. Recently, Cahuapaza-Gutierrez et al. estimated an incidence of 11.2 per 1000 patients, with a mortality rate of 7.5%, consistent with findings from the U.S. NIS study, while still confirming an overall favorable prognosis for the majority of patients [35]. Although comparative data across subtypes remain limited, available evidence suggests that type III KS may represent the highest-risk phenotype, with mortality rates reported up to 18%, substantially exceeding those observed in most mixed KS cohorts [33]. This excess risk is likely related to the occurrence of acute stent thrombosis, a condition characterized by abrupt coronary occlusion, extensive myocardial ischemia, and a high incidence of cardiogenic shock, malignant ventricular arrhythmias, and sudden cardiac death [46].

The first pediatric case of KS was reported in 2009 [47]. Pediatric KS remains exceptional and is generally associated with a favorable prognosis, with most patients recovering completely and showing preserved left ventricular function at discharge [31]. Although no pediatric deaths had initially been reported [31,48], a recent fatal case following ceftriaxone administration has been described, with death mainly attributed to severe sepsis and multiorgan failure rather than KS itself [49].

## 2. General Pathophysiology, Immunology, and Triggers

The pathophysiology of KS results from a complex interaction between the triggering allergen and cells of the innate and adaptive immune systems, leading to activation of mast cells and platelets and the release of multiple pro-inflammatory mediators. Although the upstream pathways are heterogeneous, they converge on a common final effector mechanism responsible for coronary vasomotor dysfunction and myocardial injury [18].

Mast cells are the key effector cells in KS: derived from CD34+ bone marrow precursors, they migrate to peripheral tissues, including the heart, where their density increases in the presence of coronary artery disease, and release inflammatory mediators following activation through multiple immunological pathways [20,50,51,52,53]. The best-known pathway is IgE-mediated activation. In genetically predisposed individuals, first exposure to an allergen leads to sensitization with production of high-affinity, antigen-specific IgE antibodies. These IgE molecules bind to the FcεRI receptor on mast-cell membranes and remain bound until a subsequent encounter with the same allergen, which then cross-links receptor-bound IgE and triggers mast-cell activation and degranulation [54].

Several IgE-independent pathways have also been identified, explaining why KS may occur in non-atopic individuals or during first exposure to an allergen [50,52,54,55]. These include complement-mediated activation through anaphylatoxins (C1q, C3a, C4, C5a and factor B), IgG-dependent activation via FcγRI in the presence of interferon-γ, and direct stimulation of the MRGPRX2 receptor by peptide-structured drugs, opioids, fluoroquinolones, vancomycin, neuromuscular blocking agents, and several anesthetics [56,57,58,59,60]. Complement-mediated reactions may be attenuated by premedication with antihistamines and corticosteroids and by reducing infusion rates for intravenous agents [52]. Regardless of the initiating mechanism, all pathways converge on an increase in intracellular Ca^2+^, triggering mast-cell degranulation and the release of preformed and newly synthesized inflammatory mediators, including histamine, tryptase, platelet-activating factor (PAF), cytokines (IL-4, IL-6, TNF-α), prostaglandins, leukotrienes, chemokines, proteolytic enzymes, and hematopoietic factors [61,62]. Collectively, these mediators promote coronary vasomotor dysfunction while also inducing increased vascular permeability, tissue oedema, leukocyte recruitment, bronchoconstriction, urticaria, angio-oedema, and hypotension [63,64].

The coronary circulation represents one of the principal cardiovascular targets of mast-cell activation. Even in healthy individuals, mast cells are distributed within the myocardium, perivascular connective tissue, and the intima and adventitia of coronary arteries, where they express high-affinity IgE receptors [65]. In type I KS, the combined action of histamine, cytokines, chemokines, tryptase, and PAF produces a coronary vasoconstrictor effect by inducing contraction of smooth-muscle cells in the media of coronary arteries, resulting in luminal narrowing and reduced myocardial blood flow. Vasoconstriction may be mediated directly by histamine via H_1_ and H_2_ receptors, by angiotensin II (whose generation from angiotensin I is promoted by chymases), or by leukotrienes, thromboxanes, and PAF [1,52,65]. Histamine may exert apparently paradoxical coronary effects because different receptor subtypes mediate divergent vascular responses. Experimental and human coronary artery studies indicate that H_1_ receptor activation predominantly induces coronary smooth-muscle contraction and vasoconstriction, whereas H_2_ receptor activation mediates relaxation, usually to a lesser extent. In the setting of massive mast-cell degranulation, this receptor imbalance may favor focal or diffuse coronary spasm, while concomitant systemic vasodilation and increased vascular permeability contribute to hypotension and reduced coronary perfusion pressure. This dual H_1_/H_2_ biology helps explain how the same mediator may simultaneously promote allergic shock and coronary vasospasm [66]. However, available human studies describe regional differences along the coronary tree, especially greater histamine sensitivity in proximal epicardial segments, rather than a specific coronary predominance [66,67,68]. Mast cells are present in both normal coronary arteries and the adventitia of atherosclerotic plaques, where histopathological studies have demonstrated increased density, particularly within vulnerable regions prone to rupture. Their activation has been closely associated with plaque erosion and rupture, with mast-cell degranulation documented both adjacent to culprit plaques and at sites of coronary spasm, supporting a direct contribution to plaque destabilization and acute atherothrombotic complications in KS [8,10,69,70,71]. The principal mast cell-derived mediators involved in the allergic cascade of KS, together with their cellular sources, cardiovascular effects, and potential contributions to coronary vasospasm, plaque destabilization, and thrombosis, are summarized in Supplementary Table S1.

Although KS case series often suggest frequent RCA involvement, there is no definitive evidence that this artery normally contains a higher mast-cell or eosinophil density than other epicardial vessels. However, some evidence from rare eosinophilic coronary periarteritis cases demonstrates its selective eosinophil- and mast-cell-rich inflammation, raising the hypothesis that allergic coronary inflammation may occasionally show segmental or vessel-specific distribution [72,73].

In type II KS, the massive release of inflammatory mediators exerts a strong prothrombotic effect, transforming a previously “stable” plaque into an “unstable” one and predisposing it to rupture. Histamine contributes to both coronary vasospasm and thrombosis by promoting tissue factor expression, platelet activation, and platelet aggregation, thereby amplifying both vasospastic and thrombotic pathways [1,18]. Leukotrienes, PAF, IL-6, thromboxanes, tryptase, and chymase further amplify vascular inflammation and thrombosis [74,75]. In particular, tryptase and chymase activate matrix metalloproteinases within the atherosclerotic plaque, promoting degradation of extracellular matrix components and collagen fibers of the fibrous cap; this process weakens plaque stability, facilitating plaque erosion or rupture and subsequent thrombosis [10].

The combined actions of these mediators determine the different clinical phenotypes of KS, ranging from isolated coronary vasospasm (type I) to plaque rupture (type II) and stent or graft thrombosis (type III–IV). As shown in Table 2, numerous triggers have been reported [1,17,18]. In the vast majority of patients (93.7%), a triggering allergen can be identified [30]. Drugs account for approximately 44% of reported cases, with antibiotics representing the predominant class. Amoxicillin is responsible for nearly 40% of antibiotic-related cases, whereas ceftriaxone is the most frequently reported cephalosporin [30,31,32,33,35,50,76]; NSAID-induced KS generally affects younger patients, whereas contrast media-induced KS is more common in older individuals [34]. Among the numerous agents, intravenously chemotherapeutic agents warrant particular attention because of their potential to trigger immediate and severe hypersensitivity reactions [77]. Notably, contrast media-triggered KS appears to represent one of the most severe clinical phenotypes of type I KS [78], with reported rates of cardiac arrest and mortality reaching 23.1% and 7.7%, respectively, suggesting a particularly aggressive mast cell-mediated cardiovascular response. Although iodinated contrast agents remain the most commonly reported triggers, KS has also been described following gadolinium-based and ultrasound microbubble contrast agents, indicating that mast-cell-mediated coronary events represent a potential class effect of contrast-enhanced imaging rather than a complication restricted to iodinated compounds [79]. Moreover, a notable clinical paradox is that several drugs commonly used in the management of KS, including aspirin, clopidogrel, epinephrine, heparin, and corticosteroids, have themselves been reported as potential triggers of the syndrome [80]. Their use should therefore be carefully individualized, particularly in patients with a suspected or documented hypersensitivity to these agents [81].

Implanted cardiac and endovascular devices may represent a rare source of hypersensitivity. Metals and polymers used in these devices can induce immune reactions, and nickel–titanium devices have been associated with systemic hypersensitivity syndromes resembling KS; however, direct evidence linking individual device components to overt KS remains limited [82].

Beyond the allergic trigger itself, individual susceptibility may also be influenced by neuropsychological factors. Anxiety and depression have emerged as important cardiovascular risk modifiers through sustained sympathetic activation, autonomic imbalance, endothelial dysfunction, platelet activation, oxidative stress, and chronic low-grade inflammation [83]. Mechanistically, psychological stress may also directly interact with mast-cell signaling: corticotropin-releasing hormone (CRH) can activate cardiac mast cells through specific surface receptors, while neurotensin, released together with CRH from peripheral nerves, may amplify this response and promote IL-6 release, providing a potential neuroimmune link between psychological stress, mast-cell activation, and coronary inflammation [70,84]. These mechanisms overlap with those implicated in KS, suggesting that psychological comorbidities may lower the threshold for allergic coronary vasomotor dysfunction in susceptible individuals. Moreover, the growing recognition of the heart–brain–vascular axis, exemplified by Takotsubo syndrome and the recently proposed ATAK complex, further supports the concept that inflammatory, autonomic, psychological, and catecholamine-mediated pathways may converge to determine cardiovascular vulnerability [83,85]. Although direct evidence in KS remains limited, this integrated neuro-immuno-cardiovascular model provides a biologically plausible framework that warrants further investigation.

## 3. Clinical Presentation and Diagnosis

### 3.1. Risk Factors, Signs and Symptoms

The vast majority of patients have no comorbidities [42]. Across all KS subtypes, at least one conventional cardiovascular risk factor (hypertension, diabetes mellitus, dyslipidemia, or smoking) is reported in 11–30% of patients [29,30], although this proportion paradoxically reaches up to 46% in KS type I [36]. Hypertension is the most frequently reported comorbidity [35,42]. Only 10–31% of patients have a previous history of allergy, often involving the same triggering allergen, underscoring the importance of a meticulous medical history while highlighting that KS remains largely unpredictable and currently not preventable [29,30,32,42,50]. Whether the prevalence of allergic history differs according to sex remains unknown, as sex-stratified data are currently lacking.

Diagnosis is based on symptoms, clinical signs, laboratory tests, ECG, echocardiography and coronary angiography. Clinical manifestations reflect the coexistence of systemic hypersensitivity and acute myocardial ischemia. Allergic features include erythema, urticaria, pruritus, angio-oedema, dyspnea, bronchospasm, hypotension, gastrointestinal symptoms, and, in severe cases, anaphylaxis. Cardiovascular manifestations include chest pain, dyspnea, diaphoresis, palpitations, syncope, malignant arrhythmias, and cardiac arrest. Although KS is classically defined by signs of allergic reaction, the absence of cutaneous or mucosal manifestations should not exclude the diagnosis [86,87,88,89]. Consistently, skin manifestations are absent in a substantial proportion of patients, with rash, urticaria, and pruritus reported only up to 30% of adult cases and in 45% of pediatric patients [31]. Instead, mucosal or soft-tissue involvement, including angioedema and peripheral oedema, has been observed in 15.3–24% of patients [15,31,34,38,44].

Cases of type II KS have also been reported in the complete absence of overt mucocutaneous allergic manifestations, highlighting that the lack of typical signs does not exclude an underlying hypersensitivity-mediated coronary event [90]. Even when present, allergic manifestations may be remarkably subtle, occasionally being limited to localized erythema or pruritus at the site of exposure, particularly following Hymenoptera stings [91,92]. Prospective data suggest that mast-cell activation may induce coronary vasomotor dysfunction even in the absence of overt anaphylaxis, supporting the concept of a continuous cardio-allergic spectrum [14].

Growing evidence indicates that cardiovascular symptoms may predominate or even represent the sole clinical manifestation. In type I KS, only one-third of patients presented with the combination of chest pain and rash [36]. Chest pain is the predominant presenting symptom, being reported in 59.1–86.8% of patients. Respiratory symptoms are also common, with dyspnea occurring in 30.6–52.4% of patients, while palpitations have been described in approximately 48%. Importantly, hemodynamic instability is not uncommon, with hypotension occurring in up to 70% and anaphylactic shock reported in up to 15–33% [16,29,30,32,35,36,42]. The wide variability in symptom frequency across studies reflects differences in study design, patient selection, and reporting practices. These findings emphasize the heterogeneous clinical spectrum of KS and explain, at least in part, its persistent under-recognition in routine clinical practice. Accordingly, clinicians should maintain a high index of suspicion whenever ACS occurs in temporal association with a potential allergic trigger, even in the absence of overt allergic manifestations.

Current evidence suggests that type I KS should no longer be regarded exclusively as a complication of overt anaphylaxis but rather as part of a continuous cardio-allergic spectrum in which even subclinical mast-cell activation may precipitate coronary vasomotor dysfunction and ACS.

### 3.2. ECG, Arrhythmias and Echocardiographic Findings

Electrocardiographic abnormalities are very common in KS, although their prevalence varies according to study design, subtype distribution, and timing of ECG acquisition. Ischemic ECG changes are reported in the vast majority of adult and pediatric patients, spanning a broad spectrum from transient ST-elevation myocardial infarction (STEMI)-like patterns to non-specific repolarization abnormalities, conduction disturbances, malignant arrhythmias, and, occasionally, normal tracings. These findings underscore the importance of serial ECG assessment whenever ACS develops following exposure to a plausible allergic trigger. ST-segment elevation represents the dominant ECG pattern, occurring in approximately 43–76% of mixed KS cohorts and in about 71% of type I and 82% of type III KS. Inferior ST-segment elevation predominates (66.9%), followed by anterior (16.5%), anterolateral (7.5%), lateral (5.3%), and diffuse (2.3%) patterns, although prospective allergy-selected cohorts show a more heterogeneous distribution with anterior STEMI as the predominant pattern. ST-segment depression, T-wave inversion, and non-specific ST–T abnormalities occur less frequently (up to 38%), whereas completely normal ECGs are uncommon (less than 5%). Arrhythmias and conduction disturbances include sinus tachycardia (33.3%), bradycardia (10%), atrial fibrillation (2.9–4.8%), ventricular ectopy, ventricular tachycardia (1.7%) and atrioventricular block (5.9%) [15,28,29,30,31,32,33,34,38,46,87,93]. Cardiac arrest may occur with ventricular fibrillation (10%), asystole, or pulseless electrical activity (PEA), the latter being particularly associated with contrast media-triggered KS [20,78,94,95,96,97,98,99]. The relatively frequent occurrence of PEA and asystole in contrast-induced KS suggests that cardiac arrest may not exclusively result from malignant ventricular arrhythmias but also from profound vasodilatory shock, diffuse coronary vasospasm, and severe microvascular dysfunction associated with systemic anaphylaxis [79].

Echocardiographic abnormalities are common and often reversible, reflecting transient myocardial ischemia rather than permanent structural injury. Regional wall-motion abnormalities are observed in approximately 50–79% of patients, whereas normal echocardiographic findings are reported in 42–50%. Left ventricular systolic dysfunction is usually mild; reduced left ventricular ejection fraction (LVEF) < 50% occurs in approximately 17% of patients, whereas preserved LVEF is maintained in nearly 72%. Recovery of regional wall-motion abnormalities is documented in up to 92.9% of cases within days or weeks, while 95% of pediatric patients show normal LVEF at discharge. Persistent left ventricular dysfunction appears uncommon, affecting approximately 7% of patients [29,31,32,42].

### 3.3. Biochemical and Laboratory Assessment

Serial measurement of cardiomyocyte necrosis markers, particularly high-sensitivity cardiac troponin and CK-MB, is essential to detect myocardial injury and distinguish allergic unstable angina from allergic myocardial infarction. Cardiac biomarker elevation is reported in approximately 60.6–78.1% of adults and 85% of pediatric patients. Accordingly, normal biomarker levels do not exclude type I KS, particularly when coronary vasospasm is transient or ECG abnormalities resolve rapidly [29,30,31,32,35]. These tests should be complemented by standard ACS blood work, such as complete blood count, lipid profile, glucose, and BNP [100].

Serum histamine is rarely measured due to its kinetics: the peak occurs very early (1–5 min) and its half-life is very short (≈8–30 min), making false-negative results likely if sampling is not immediate (ideal window 10–60 min) or if opioids such as morphine have been administered [1,18,101,102]. A urinary metabolite, 24 h methylhistamine, can also be measured [103].

Serum tryptase is the most specific laboratory marker of mast-cell activation and plays a central role in the diagnosis of KS [1,18]; although small amounts are present in basophils, circulating tryptase in KS derives almost exclusively from activated mast cells [104]. Peak levels occur 1–2 h after symptom onset, with a half-life of approximately 90 min and return to baseline within 24 h [105,106]. Accordingly, blood sampling should ideally be performed within 30 min to 2 h (and no later than 4 h) to minimize false-negative results [107]. Normal serum tryptase is <11.5 ng/mL, whereas mast-cell activation is supported by concentrations >11.5–15 ng/mL or, preferably, by the validated dynamic criterion of a ≥20% + 2 ng/mL increase from baseline, which provides greater diagnostic accuracy than a fixed cut-off [50,108,109]. Tryptase may remain normal (false negative) in food-induced anaphylaxis or when sampling is delayed (after 12–24 h); therefore, normal values do not exclude KS [109,110]. There may also be discordant patterns between histamine and tryptase, with elevation of only one mediator [18,111]. Despite these limitations, elevated tryptase levels have been reported in approximately 80.6–87.7% of tested patients and in all individuals assessed in a contemporary real-world cohort [29,30,32,42]. Given these kinetic limitations, early blood sampling should be incorporated into the initial evaluation of all patients with suspected allergic ACS, ideally before administration of anti-allergic therapy whenever clinically feasible. Beyond its diagnostic role, tryptase may also have pathophysiological relevance, as it can contribute to plaque erosion or rupture through matrix metalloproteinase activation and may amplify coronary inflammation by inducing MCP-1 and IL-8 expression [10,35].

Total and allergen-specific IgE may provide complementary information but have different clinical implications. Elevated total IgE may support an underlying allergic predisposition but has limited specificity and cannot establish a causal relationship with the acute coronary event. Conversely, allergen-specific IgE testing may help identify sensitization to a suspected trigger and can therefore contribute to the subsequent allergological work-up and prevention of re-exposure. However, neither normal total nor negative specific IgE excludes KS, since mast-cell activation may occur through both IgE-dependent and IgE-independent pathways [1,18,50]. Elevated total IgE levels are reported in approximately 72–75.7% of tested patients, whereas peripheral eosinophilia occurs in 46.6–58.3% [29,32,33,42]. Peripheral eosinophilia represents another supportive but non-specific finding. Its absence does not exclude KS, and peripheral eosinophil counts may not necessarily reflect tissue-level allergic inflammation. Nevertheless, eosinophils may have pathophysiological relevance, particularly in thrombotic forms of KS, in which eosinophilic infiltration has been demonstrated within coronary thrombi [112]. Thus, both IgE and eosinophil measurements should be interpreted as complementary and supportive findings within the overall clinical, biochemical, and angiographic context rather than as standalone diagnostic biomarkers [33]. Other inflammatory mediators, including leukotrienes and thromboxanes, have limited discriminatory value because they may also increase during conventional ACS [103].

### 3.4. Coronary Angiography

In patients presenting with ST-segment elevation and suspected ACS, coronary angiography should be performed according to the 2023 ESC Guidelines for ACS (Class I, Level A) [100]. Accordingly, coronary angiography is undertaken in the majority of patients with suspected KS, although its use varies considerably across studies, ranging from approximately 36% in older cohorts to 80–98.5% in contemporary series [32,33,34,35]. Angiographic findings are highly heterogeneous and largely reflect the underlying KS subtype. Angiographically normal or non-obstructive coronary arteries are reported in approximately 42–55% of cases, whereas angiographic abnormalities are observed in 42–58%. Critical coronary stenosis or atherothrombotic occlusion is identified in approximately 32–42% of patients and mainly associated with type II and type III KS [30,34,35].

Despite coronary vasospasm being the hallmark mechanism of type I KS, epicardial spasm is directly visualized in a minority of cases, only 10–16% of angiograms [30,35]. Regarding vessel distribution, earlier studies suggested a predominance of RCA involvement, largely inferred from the high frequency of inferior ST-segment elevation rather than from direct angiographic evidence [29,35]. However, more comprehensive angiographic analyses indicate a more balanced distribution, with involvement of the LAD artery in 27.3%, right coronary artery in 26.5%, left circumflex artery in 12.2%, and left main coronary artery in 2.5% of cases [30]. These findings suggest that virtually any epicardial coronary territory may be affected.

Whether all suspected KS patients should undergo emergent coronary angiography remains an open question. Given that up to 72% have type I, with an excellent prognosis and transient vasospasm as the central mechanism, angiography is often primarily diagnostic. On the other hand, in some type I KS cases, intracoronary vasodilators are required to relieve spasm, and in type II to IV KS, angiography assumes a life-saving therapeutic role. Although not systematically investigated, in suspected type I, a “watchful waiting” approach after anti-allergic and empirical coronary vasodilatation treatment, monitoring for regression of ECG changes and symptoms as an indirect sign of spasm resolution, especially if rapid clinical improvement is observed, may be a reasonable strategy with good outcomes and no complications, as reported in multiple descriptions [25,113,114,115,116]. Coronary angiography may therefore be reserved for cases refractory to medical therapy. The optimal safe observation window, however, is not clearly defined.

This conservative approach strategy, however, has not been prospectively validated and should not be applied to patients with suspected plaque rupture, stent thrombosis, persistent ischemia, hemodynamic instability, or a high pre-test probability of obstructive coronary artery disease, in whom urgent coronary angiography remains mandatory.

Coronary angiography remains fundamental for distinguishing among KS subtypes and enabling personalized, mechanism-based management [117]. Importantly, it should not be delayed, particularly in patients with cardiovascular risk factors, persistent symptoms, or ongoing ECG abnormalities despite empirical therapy, as this may postpone timely PCI in the presence of coronary thrombosis [92,118]. When clinically appropriate, angiography should be complemented by intracoronary imaging (optical coherence tomography—OCT or intravascular ultrasound—IVUS) and/or invasive coronary functional testing (vasoreactivity testing, coronary flow reserve—CFR, index of microvascular resistance—IMR, and fractional flow reserve—FFR) to differentiate epicardial vasospasm, CMD, flow-limiting plaque, rupture and erosion. This integrated anatomical and functional approach enables mechanism-based treatment and more accurate prognostic stratification across the full spectrum of KS [119,120,121].

Rather than relying solely on coronary anatomy, contemporary evaluation of suspected KS should integrate anatomical, functional, immunological, and clinical information to accurately define the underlying pathophysiological mechanism and guide personalized management.

### 3.5. Adjunctive Diagnostic Tests

Unlike myocarditis, in which endomyocardial biopsy may demonstrate lymphocytic, eosinophilic, or giant-cell inflammatory infiltrates, endomyocardial biopsy is typically unremarkable in KS [122]. Histopathological examination of coronary tissue is generally limited to post-mortem studies but is highly specific, demonstrating eosinophilic and/or mast-cell infiltration within ruptured plaques or thrombosed coronary stents [10,123,124,125,126]. By contrast, histopathological examination of aspirated thrombotic material obtained during coronary angiography represents the diagnostic gold standard for type III KS. Demonstration of eosinophil-rich infiltrates on hematoxylin–eosin staining and/or mast cells identified by May–Grünwald–Giemsa staining within the aspirated thrombus provides definitive evidence of an allergic thrombotic mechanism. However, thrombus histology is not routinely performed and is therefore available only in a minority of patients [1,46,50].

In patients with suspected stent hypersensitivity, cutaneous patch testing may identify sensitization to metallic components of the stent platform, thereby supporting the diagnosis of type III KS and strengthening the causal link between hypersensitivity and stent thrombosis [46]. These adjunctive investigations are reserved for selected clinical scenarios but may provide definitive mechanistic confirmation when conventional clinical, angiographic, and laboratory findings are inconclusive.

Finally, artificial intelligence may emerge as a valuable tool in the management of KS, with potential applications ranging from early recognition and risk stratification to treatment guidance and longitudinal monitoring. However, current evidence remains preliminary, and further data, particularly prospective validation studies, are needed before these approaches can be reliably integrated into clinical practice [127].

## 4. Specific Clinical Phenotypes of Kounis Syndrome: From Vasomotor Dysfunction to Thrombosis

### 4.1. Type I KS: Allergic Coronary Vasomotor Dysfunction

#### 4.1.1. Definition and Current Concept

Type I KS is characterized by allergic coronary vasospasm occurring in patients with normal or near-normal epicardial coronary arteries, classically without significant cardiovascular risk factors [1,11]. Vasospastic angina (Printzmetal angina) is traditionally defined as a transient coronary vasomotor disorder characterized by reversible epicardial coronary artery constriction resulting in myocardial ischemia [128]. In the context of KS, this paradigm can be extended to encompass allergy-mediated coronary hyperreactivity, whereby mast-cell activation and the release of vasoactive and pro-inflammatory mediators precipitate dynamic epicardial vasoconstriction in the absence of fixed obstructive coronary artery disease [50].

In the first systematic review specifically dedicated to type I KS, Quetsch et al. adopted a stringent case-identification strategy combining the COronary VAsomotion Disorders International Study (COVADIS) diagnostic criteria for vasospastic angina with objective evidence of an acute allergic reaction occurring within 24 h of a recognizable trigger, supported by compatible clinical manifestations and/or laboratory markers of allergic activation [36]. According to the COVADIS criteria, vasospastic angina is classified as definitive or suspected based on nitrate-responsive angina, transient ischemic ECG changes, and/or angiographic demonstration of reversible coronary spasm (>90%) associated with chest pain and ischemic ECG abnormalities [36,129]. This approach improved diagnostic specificity and represented the first attempt to apply standardized, reproducible diagnostic criteria to type I KS, a condition historically characterized by substantial heterogeneity in case definitions and diagnostic reporting. In published literature-based reviews of case reports and case series, type I KS consistently emerges as the most prevalent clinical presentation, accounting for 43.4% to 72% of cases, and includes both the youngest and the oldest patients reported with KS [26,28,29,30,31,33,34,35]. However, these estimates derive almost exclusively from case reports and case series, as prospective studies and dedicated registries remain unavailable. In the largest retrospective single-center study to date, conducted by Dogan et al., nearly 90% of the 28 patients with KS who underwent invasive coronary angiography or coronary CT angiography were classified as type I [42]. This variability reflects the absence of dedicated registries, guideline-based diagnostic frameworks, and universally accepted diagnostic criteria, together with persistent under-recognition of the syndrome.

Although type I KS can occur across a wide age range, it most commonly affects middle-aged individuals, with available evidence demonstrating a median age of 53 years, a clear predominance among men (64%) [36], and notably, all pediatric cases reported to date have been classified in this category [31].

A further unresolved epidemiological feature of KS is the consistent male predominance reported across most observational series. This finding is particularly noteworthy because it appears to contrast with the sex distribution of other coronary vasomotor disorders, in which microvascular dysfunction and some vasospastic phenotypes are frequently reported among women. Sex-related differences are also increasingly recognized across the heterogeneous spectrum of type 2 MI, potentially involving differences in underlying coronary disease, precipitating mechanisms, comorbidities, clinical presentation and diagnostic pathways [130,131]. However, whether the male predominance observed in KS reflects biological susceptibility, differential exposure to allergic triggers and cardiovascular risk factors, differences in clinical recognition, or reporting and selection bias remains unknown. Dedicated sex-stratified studies are lacking, and the mechanisms underlying this apparent epidemiological paradox should therefore be considered an important knowledge gap in KS.

Finally, type I KS embodies a remarkable clinical paradox: although patients may present with life-threatening arrhythmias, cardiogenic shock, pulseless electrical activity (PEA), asystole, or profound hemodynamic instability, myocardial dysfunction usually resolves completely without permanent scar formation, resulting in an excellent long-term prognosis with reported mortality as low as 1.4% [16,33,79,132,133].

#### 4.1.2. Angiographic Characteristics and Coronary Invasive Assessment

From an angiographic perspective, coronary vasospasm can present in different patterns:

- Focal: a localized, relatively short segment of a single artery (focal) [77,134];
- Multifocal: multiple segments of the same vessel (multifocal) [135];
- Diffuse single vessel: most of the vessel in a relatively homogeneous manner [86,136,137,138];
- Multivessel: more than one (up to three) coronary artery simultaneously [135,139,140,141], present in 44% of these cases, suggesting a frequently diffuse vasomotor response rather than focal vessel involvement [36].

Spasm may also affect coronary venous bypass grafts along with native coronary arteries [142]. Paradoxically, despite epicardial coronary vasospasm being regarded as the defining pathophysiological substrate of type I KS, angiographic evidence of spasm during the acute phase is demonstrated in only a minority of patients (10.3–16.3%) [30,35].

In most patients, coronary angiography shows normal or near-normal coronary arteries, supporting the concept that myocardial ischemia may result either from transient vasospasm that has resolved before imaging or from coronary microvascular dysfunction (CMD), which remains largely undetectable by conventional angiography [1,17,38,93]. Therefore, absence of epicardial disease is a very frequent condition in the syndrome, observed in more than 50% in the general series [30,32].

Consistent with these observations, the first type I KS systematic review reported the absence of significant obstructive coronary artery disease in 75% of patients, whereas spontaneous or provoked coronary vasospasm was documented in 25% of those undergoing angiographic assessment, further supporting the concept that this subtype is predominantly a functional coronary disorder [36].

Type I KS may occur in patients with completely normal coronary arteries as well as in those with non-critical coronary atherosclerosis (stenosis <70%, or <50% for the left main coronary artery), in whom allergic vasospasm may transiently convert a non-obstructive lesion into a functionally significant stenosis [143,144]. Accordingly, the presence of mild coronary atherosclerosis, particularly in older patients with cardiovascular risk factors, should not automatically lead to a diagnosis of type I KS, as differentiation should primarily rely on the underlying pathophysiological mechanism (vasospasm versus plaque disruption with thrombosis) rather than on the mere presence or absence of coronary artery disease [38,145,146,147]. As previously highlighted, the absence of angiographically demonstrable epicardial vasospasm during the acute phase appears to represent the rule rather than the exception. Rather than challenging the diagnosis of type I KS, this finding broadens its pathophysiological spectrum and suggests that the absence of visible spasm may reflect several non-mutually exclusive mechanisms: predominant coronary microvascular spasm and endothelial dysfunction, spontaneous resolution of transient epicardial vasoconstriction before angiography, regression following anti-allergic and/or coronary vasodilator therapy, or the simultaneous contribution of these mechanisms [11,119,144,148,149,150,151,152,153].

Among these mechanisms, coronary microvascular spasm and dysfunction deserve particular attention. Originally hypothesized in the first description of the syndrome in 1991, this concept remained largely unexplored for decades but is now increasingly supported by evidence from multimodality imaging, including cardiac magnetic resonance (CMR) and myocardial perfusion scintigraphy [16,94,119,148]. Consequently, angiographically normal epicardial coronary arteries should not be interpreted as evidence against myocardial ischemia, as the coronary microcirculation may represent the primary site of allergic coronary dysfunction [128]. A complementary pathogenic mechanism may involve transient severe vasospastic obstruction followed by ischemia–reperfusion injury, leading to osmotic overload-induced myocyte swelling and progressive extension of injury from the initially affected region toward the epicardium [154]. Accordingly, the longer the interval between symptom onset and coronary angiography, particularly after early administration of anti-allergic and vasodilator therapy, the greater the likelihood of finding angiographically normal coronary arteries [155].

**Figure 2:**
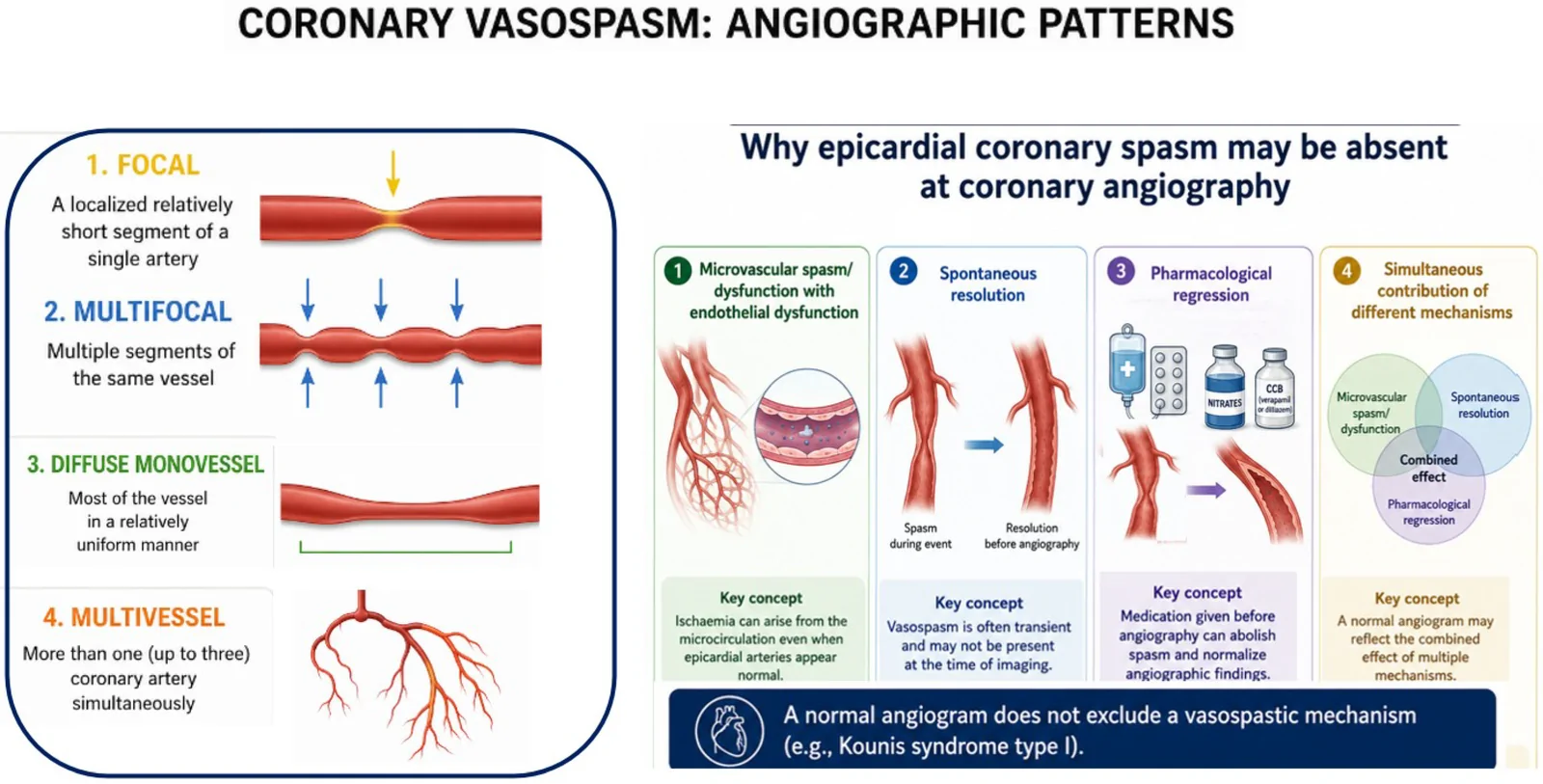
Angiographic patterns of epicardial coronary vasospasm and mechanisms underlying the absence of angiographically visible spasm. Epicardial vasospasm may present as focal, multifocal, diffuse monovessel, or multivessel constriction. A normal coronary angiogram does not exclude a vasospastic mechanism, as spasm may be confined to the coronary microcirculation, may have resolved spontaneously before imaging, may regress after administration of anti-allergic or vasodilator therapy, or may reflect the simultaneous contribution of multiple mechanisms. These concepts are particularly relevant in patients with suspected Kounis syndrome type I.

Angiographic patterns and mechanisms underlying the absence of visible spasm are summarized in Figure 2.

Coronary angiography is often not performed because chest pain, ECG abnormalities, and biomarker elevation may resolve rapidly following anti-allergic and/or empirical vasodilator therapy [25]. Consequently, type I KS is frequently diagnosed retrospectively, with transient allergic coronary vasospasm inferred from the temporal association between an allergic reaction and reversible ACS-like manifestations rather than directly documented angiographically [113,152,156,157]. Many cardiologists therefore opt for a conservative strategy considering the complete clinical remission [156,158].

The role of coronary functional testing in type I KS remains largely unexplored but may prove pivotal for refining its diagnosis and pathophysiological classification. Most reported cases are currently diagnosed on the basis of an allergic trigger, transient ischemic symptoms or ECG abnormalities, elevated cardiac biomarkers, and the absence of obstructive coronary artery disease. However, this diagnostic approach probably underestimates the full spectrum of coronary vasomotor dysfunction.

Once the allergic reaction has resolved and the patient is hemodynamically stable, intracoronary acetylcholine (ACh) or ergonovine provocation testing could identify inducible epicardial spasm while simultaneously distinguishing epicardial from microvascular vasospasm, thereby providing a direct pathophysiological rather than purely exclusionary diagnosis [119,136,159]. In addition, invasive assessment of CFR and the IMR may reveal concomitant CMD, a well-established mechanism in patients with ischemia and non-obstructive coronary arteries (INOCA) [128]. Particularly compelling is the observation that allergic coronary vasomotor dysfunction may coexist with CMD and transient Takotsubo-like abnormalities despite angiographically normal coronary arteries, supporting the concept that allergic mediator release may simultaneously affect both epicardial conductance vessels and the coronary microcirculation [119]. Although current evidence is limited and standardized protocols are lacking, coronary functional testing has the potential to substantially improve diagnostic accuracy, refine phenotypic classification, and integrate type I KS into contemporary invasive coronary physiology pathways rather than leaving it as a diagnosis of exclusion.

Future diagnostic algorithms should aim to establish a pathophysiological diagnosis based on coronary vasomotor and microvascular assessment rather than relying solely on the absence of obstructive coronary artery disease.

#### 4.1.3. Electrocardiographic Characteristics

The electrocardiographic (ECG) manifestations of type I KS are highly heterogeneous and reflect the transient nature of allergic coronary vasospasm. The ECG plays a pivotal diagnostic role by capturing the dynamic electrical consequences of transient coronary vasomotor dysfunction occurring during the acute allergic reaction. However, interpretation of the available electrocardiographic evidence is complicated by a recurring subtype extrapolation bias. Because most published series combine all KS subtypes, aggregate ECG findings are frequently attributed to individual variants despite their distinct pathophysiological mechanisms.

Among the earliest reviews, STEMI represented the most frequently reported ECG presentation, accounting for approximately 76% of cases, with nearly two-thirds involving the inferior leads [29]. Consequently, inferior ST-segment elevation has traditionally been regarded as the typical ECG pattern of KS. However, this interpretation warrants caution because it derives largely from mixed KS populations and is not supported by angiographic evidence [30]. Inferior ST-segment elevation may reflect right coronary artery (RCA) involvement but can also occur with left circumflex or multivessel spasm, or anatomical variants such as a wrap-around left anterior descending (LAD) artery [160,161,162]. Type I KS-specific evidence has subsequently emerged from two prospective CMR studies and a dedicated systematic review. In the prospective study by Akoz et al., ST-segment elevation was observed in only 43% of patients, considerably lower than in earlier pooled analyses; ECG abnormalities were heterogeneous, with anterior ST-segment elevation predominating over inferior involvement, whereas non-specific ST–T abnormalities were also common. Interestingly, almost one-third of anterior ST-segment elevations measured <2 mm, suggesting that conventional STEMI thresholds for precordial leads V2-V3 may underestimate myocardial ischemia related to allergic vasomotor dysfunction [16,100]. Comparable findings were reported by Okur et al., who identified ST-segment elevation in only 34% of patients with type I KS, further supporting a broader and less stereotyped electrocardiographic spectrum than previously recognized [132].

More recently, Quetsch et al. provided the first literature-based review exclusively dedicated to type I KS. Electrocardiographic data were available for 380 patients, among whom ST-segment elevation was observed in approximately 70%, making it the predominant electrocardiographic manifestation of type I KS [36]. The discrepancy in incidence with CMR cohorts is likely explained by publication bias, as striking STEMI presentations are more likely to be reported than milder cases characterized by transient ischemic changes, non-specific ST–T abnormalities, or even normal ECGs. Consequently, although ST-segment elevation remains the most frequently reported ECG manifestation of type I KS, its true prevalence is probably influenced by study design and case selection.

Patients with type I KS may also present with STEMI-equivalent electrocardiographic signs, including “shark-fin” and de Winter patterns, which are usually considered hallmarks of proximal LAD occlusion and extensive myocardial ischemia, or with the “south African flag sign”, usually indicative of anterolateral ischemia due to first diagonal branch or ramus intermedius involvement [91,163,164,165,166]. Another possible electrocardiographic pattern of presentation is characterized by widespread ST-segment depression across multiple leads with concomitant ST-segment elevation in aVR, indicative of diffuse subendocardial ischemia, even in the absence of significant left main or proximal LAD stenosis [167]. The occurrence of these high-risk ECG phenotypes in patients with angiographically normal or non-obstructive coronary arteries reinforces the concept that severe allergic coronary vasomotor dysfunction can reproduce the full spectrum of ischemic electrical manifestations traditionally attributed to atherothrombotic STEMI.

#### 4.1.4. Advanced Imaging: CMR, CCTA, Nuclear Imaging

CMR has emerged as the most informative non-invasive imaging modality for evaluating myocardial injury in type I KS, particularly in patients with non-obstructive coronary arteries. Beyond documenting myocardial oedema and transient ischemic injury, CMR plays a pivotal role in distinguishing type I KS from other MINOCA etiologies, especially myocarditis and Takotsubo syndrome [100,168,169].

Available evidence suggests that myocardial involvement in type I KS is not uniform.

The first prospective study, conducted by Akoz et al., evaluated 21 patients presenting with allergic reactions and clinical features consistent with type I KS who underwent CMR within 24 h. CMR showed myocardial oedema and early gadolinium enhancement in the absence of late gadolinium enhancement (LGE), a pattern consistent with acute reversible ischemic injury. Regional wall-motion abnormalities were observed in a subset of patients, whereas myocardial abnormalities involved the interventricular septum, left ventricular free wall and apex [16]. This predominance challenges the traditional concept of preferential RCA involvement derived from earlier ECG-based observations and suggests a more complex spatial distribution of allergic myocardial injury [29], with the frequent involvement of the interventricular septum potentially reflecting a greater vulnerability of its microcirculation. Interestingly, even in patients presenting with ST-segment elevation, CMR abnormalities remained confined to the subendocardium and were also detected in patients with a normal ECG, underscoring the superior sensitivity of CMR for identifying myocardial involvement in type I KS [16].

Similar findings were reported by Okur et al. in a prospective study including 26 patients with clinical features consistent with type I KS. CMR consistently demonstrated subendocardial myocardial involvement, predominantly affecting the interventricular septum in about half of cases, followed by the left ventricular free wall and apex, without evidence of LGE, further supporting the concept of reversible myocardial injury rather than irreversible necrosis [132].

Conversely, completely normal CMR findings, including preserved native T1 and T2 mapping values and the absence of LGE, have also been reported [170], suggesting that myocardial involvement in type I KS is heterogeneous and that its imaging phenotype remains incompletely characterized, warranting further investigation.

Current ESC guidelines recommend early CMR in patients with MINOCA, as it establishes the underlying diagnosis in up to 87% of cases [100]. In type I KS, CMR is particularly valuable for detecting myocardial oedema and transient regional wall-motion abnormalities, thereby documenting ischemic myocardial injury [171].

Coronary computed tomography angiography (CCTA) is emerging as a valuable non-invasive alternative to invasive coronary angiography in selected patients with suspected type I KS, particularly younger individuals with a low pre-test probability of obstructive coronary artery disease. Although CCTA cannot directly visualize transient coronary vasospasm, its principal role is to exclude atherosclerotic coronary artery disease and other structural causes of myocardial ischemia; demonstration of normal coronary anatomy, interpreted together with allergic symptoms, biomarker abnormalities, ECG changes, and complementary CMR findings, substantially increases diagnostic confidence [16,33,42,132]. As CCTA becomes increasingly integrated into contemporary MINOCA pathways, its role in the diagnostic work-up of suspected type I KS in low-risk and hemodynamically stable patients, in whom an invasive strategy is not immediately required, is likely to expand [172].

Evidence on nuclear imaging in type I KS remains limited to isolated case reports. Available studies employing thallium-201 and I-123 BMIPP (β-Methyl-p-Iodophenyl-Pentadecanoic Acid) single-photon emission computed tomography (SPECT) consistently demonstrated perfusion and metabolic abnormalities despite angiographically normal coronary arteries, providing objective evidence of myocardial ischemia and supporting the concept of CMD [119,148]. Nuclear imaging may provide valuable complementary functional information, particularly when CMR is contraindicated or unavailable.

Takotsubo syndrome represents both an important differential diagnosis and a potential overlapping phenotype of type I KS. This interaction has been conceptualized as the ATAK complex (adrenaline, Takotsubo, anaphylaxis, Kounis syndrome), whereby anaphylaxis-related inflammatory mediators, endogenous catecholamine release, and exogenous adrenaline may synergistically promote epicardial or microvascular spasm, endothelial dysfunction, and direct catecholamine-mediated myocardial stunning [85,173,174]. Consequently, distinguishing type I KS from Takotsubo syndrome during the acute phase may be challenging. In this setting, multimodality imaging, complemented by invasive coronary functional testing in selected patients, can be essential to differentiate true Takotsubo syndrome from KS-related myocardial stunning and to identify cases in which the two entities coexist [119,174].

#### 4.1.5. A Distinct ACS-MINOCA Endotype Ready to Be Recognized in Future Guidelines

In the contemporary classification of ACS, type I KS should be regarded as a distinct allergic vasospastic endotype of MINOCA, driven by hypersensitivity-mediated coronary vasomotor dysfunction rather than atherosclerotic plaque rupture or fixed obstructive coronary artery disease [175]. MINOCA encompasses patients with evidence of myocardial infarction and non-obstructive coronary arteries (<50% stenosis). Rather than representing a final diagnosis, it constitutes a working diagnosis that initiates a structured diagnostic pathway aimed at excluding non-ischemic cardiac disorders (e.g., Takotsubo syndrome, myocarditis, cardiomyopathies) and extracardiac causes of myocardial injury, followed by etiological characterization using multimodality imaging, particularly CMR and, when appropriate, intracoronary imaging with optical coherence tomography (OCT) and/or intravascular ultrasound (IVUS) [100,168,176]. Comprehensive coronary functional assessment further refines the diagnosis by evaluating epicardial physiology (e.g., FFR), coronary microvascular function (e.g., coronary flow reserve and indices of microvascular resistance), and vasoreactivity through acetylcholine provocation testing, according to the contemporary stepwise “full physiology” approach [128,176,177]. Contemporary ESC and ACC/AHA ACS guidelines recognize coronary vasospasm and CMD as potential mechanisms of myocardial infarction and frame MINOCA as a working diagnosis requiring further etiological clarification. However, neither document specifically recognizes KS or allergic myocardial infarction as a distinct allergic vasospastic ACS/MINOCA endotype, leaving this unique pathophysiological entity largely unaddressed within current diagnostic frameworks [100,178,179]. Although not yet formally incorporated into current guideline documents, type I KS can be reasonably positioned within the contemporary MINOCA framework as a form of type 2 MI caused by hypersensitivity-mediated coronary vasomotor dysfunction. More specifically, it belongs to the subgroup characterized by reduced myocardial oxygen supply, in which epicardial coronary spasm and CMD represent the predominant mechanisms [100,168,175].

Type 2 MI represents a heterogeneous pathophysiological entity encompassing multiple conditions that disrupt the balance between myocardial oxygen supply and demand in the absence of acute atherothrombosis. These include systemic precipitants that reduce oxygen supply, such as anemia, hypoxemia, and hypotension, as well as cardiac triggers that predominantly increase myocardial oxygen demand, including sustained tachyarrhythmias and severe hypertension. In contrast, type I KS represents a mechanism-specific, primarily supply-side form of type 2 MI, in which an acute hypersensitivity reaction directly impairs coronary blood flow through epicardial and/or microvascular vasomotor dysfunction [130].

Formal recognition of type I KS as a distinct allergic vasospastic MINOCA endotype would facilitate a more standardized diagnostic pathway, encourage the systematic use of multimodality imaging and coronary functional testing, increase clinicians’ awareness, and ultimately improve recognition of a condition that remains substantially underdiagnosed.

### 4.2. Other Kounis Syndrome Subtypes

#### 4.2.1. Type II KS: Allergic Atherothrombosis

The allergic insult triggers a biochemical cascade leading to destabilization and rupture/erosion of an atherosclerotic plaque, with subsequent thrombosis and subtotal/total coronary occlusion, resulting in type 1 MI [1,180]. Patients have one or more cardiovascular risk factors and pre-existing coronary artery disease. The prevalence of type II KS ranges from 20.3% to 27.2% [29,30,32,33,35], and in the general series coronary angiography demonstrates stenosis or occlusion in 32% and 29% of cases, respectively [35]. The only available outcome datum reports no mortality [33].

#### 4.2.2. Type III KS: Coronary Stent Thrombosis (IIIa) and Restenosis (IIIb)

Allergic stent thrombosis was first described by Kogias and colleagues and categorized by Biteker in 2010 [181,182]; later in 2017, both Ito and Biteker defined two subgroups [183,184]:

- IIIa: allergic stent thrombosis, due to an external allergen or hypersensitivity to stent components (metal strut, polymer, drug);
- IIIb: allergic stent restenosis, due to stent hypersensitivity.

This is the least frequent subtype, with a prevalence of 5.1–14.1% [29,30,31,33,35]. Affected patients are older, predominantly male, more frequently present with STEMI, and exhibit the highest reported mortality rate (18%) among KS subtypes [33,46].

#### 4.2.3. Type IV KS: Aorto-Coronary Bypass Graft Thrombosis

In 2013, Dazy proposed a potential type IV KS, due to thrombosis of a conduit used for coronary artery bypass grafting. He described an inferior wall MI occurring during an aspirin desensitisation protocol, which accidentally induced a reaction promptly treated with steroids, antihistamines and adrenaline, after which the patient nonetheless developed proximal thrombosis of a venous graft (great saphenous vein) to the posterior descending artery, while the other two bypasses (unspecified type) remained patent [185]. Additional reports include bypass graft thrombosis with cardiac arrest after anaphylaxis and adrenaline administration [186], and stent thrombosis within a venous graft [187]. It remains unclear whether graft vasospasm should be classified as type I or type IV, which would then encompass both graft thrombosis and graft spasm [142]. No cases of thrombotic KS involving an arterial bypass conduit have been reported to date, suggesting that this pathophysiological expression may be associated exclusively with venous grafts due to different biological vulnerability [39].

#### 4.2.4. Allergic Acute Coronary Syndrome: MI vs. Unstable Angina

The most recent classification has proposed subdividing all variants into two further categories based on a biochemical basis:

- allergic unstable angina: normal levels of myocyte-necrosis biomarkers;
- allergic myocardial infarction: elevated necrosis markers.

According to this subclassification, for each subtype should be specified whether a true MI (myocyte necrosis) or only unstable angina (without myocyte necrosis) occurred [38]. However, there is currently no evidence supporting a distinct prognosis between these two clinical presentations.

**Figure 3:**
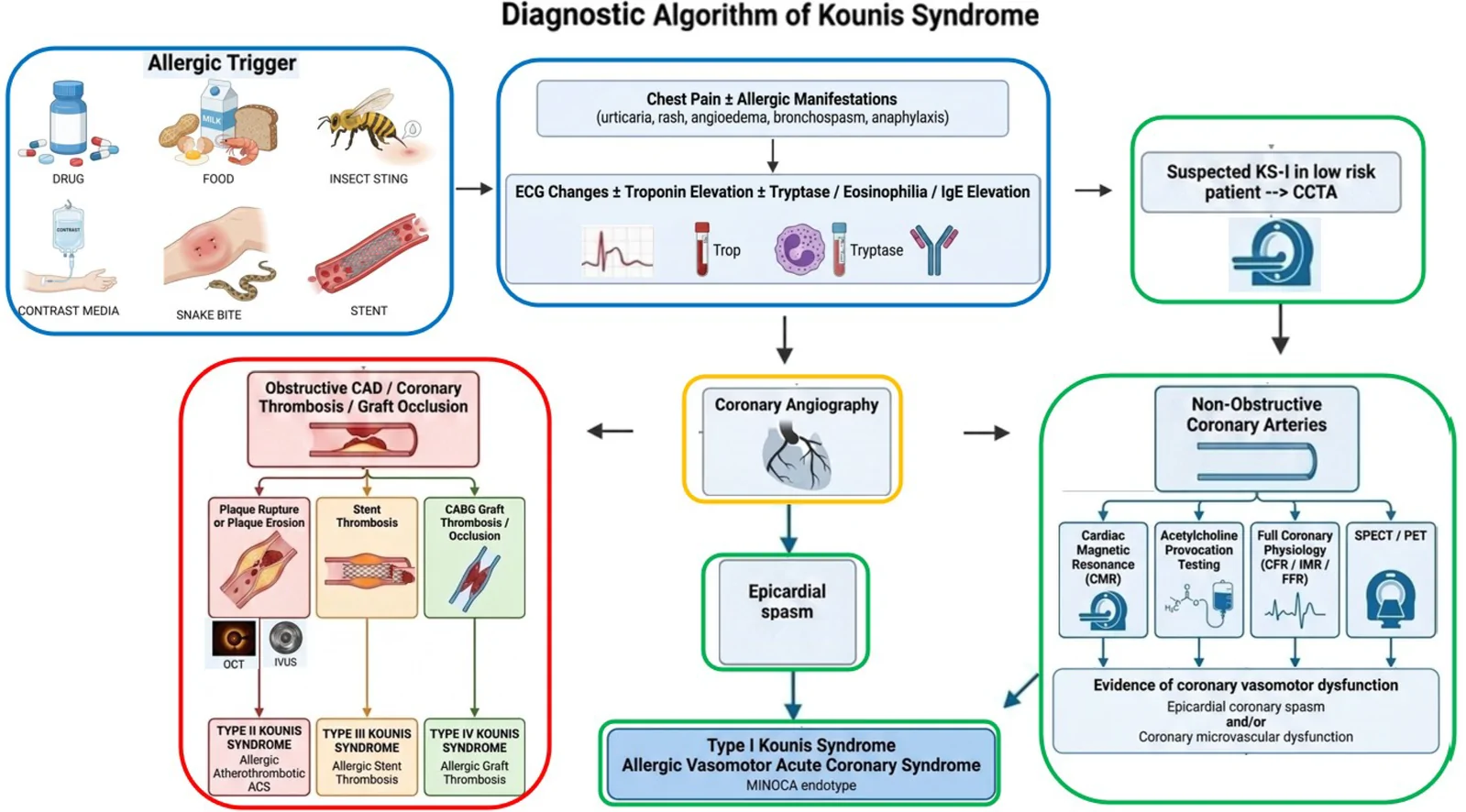
Comprehensive diagnostic algorithm for Kounis syndrome (KS). Starting from the identification of an allergic or hypersensitivity trigger associated with ACS, the algorithm guides the diagnostic work-up toward the appropriate KS subtype. Coronary angiography, or CCTA in low-risk patients, represents the cornerstone of anatomical assessment, with additional non-invasive imaging modalities, including CMR or SPECT, and, in selected cases, invasive coronary functional testing, potentially being required for a definitive diagnosis of type I KS, by identifying epicardial coronary vasospasm and/or CMD. Abbreviations: ACS, acute coronary syndrome; CABG, coronary artery bypass graft; CAD, coronary artery disease; CCTA, coronary computed tomography angiography; CFR, coronary flow reserve; CMD, coronary microvascular dysfunction; CMR, cardiac magnetic resonance; ECG, electrocardiographic; FFR, fractional flow reserve; IgE, immunoglobulin E; IMR, index of microvascular resistance; IVUS, intravascular ultrasound; OCT, optical coherence tomography; SPECT, single-photon emission computed tomography.

Figure 3 summarizes a comprehensive diagnostic algorithm of KS, integrating allergic trigger, clinical presentation, biomarkers, electrocardiography, coronary imaging, multimodality imaging, and invasive coronary functional assessment.

## 5. Management and Therapy

### 5.1. General Principles

To date, there are no specific treatment guidelines for the syndrome in its different variants, and most information derives from case reports and case series [188,189]. KS represents a therapeutic challenge, and management can be divided into two major components, which must be addressed simultaneously: the allergic reaction and the ACS [55]. Two key principles should always be borne in mind [1,190]:

(1) the systemic allergic reaction must be controlled as early as possible;
(2) some drugs may worsen myocardial ischemia and should be used with caution.

The therapeutic balance is intrinsically complex: anaphylaxis with profound peripheral vasodilatation often requires vasopressors, whereas coronary vasoconstriction calls for vasodilators. Moreover, several drugs used to treat ACS may exacerbate the allergic reaction (e.g., morphine, certain contrast agents), and conversely, anti-allergic treatments may have hemodynamic and myocardial consequences during coronary occlusion [191,192]. Overall, management is predominantly pharmacological, reported in up to 93.5% of cases, and combines anti-allergic and cardiovascular treatment [33].

A careful assessment of the patient’s allergic history is essential both for acute treatment and long-term prevention of KS [87]. Anti-allergic therapy is administered in approximately 70–80% of reported cases and mainly consists of corticosteroids and antihistamines, which are used in more than half of patients and even more frequently in pediatric cohorts [29,30,31,32]. Corticosteroids represent the most frequently prescribed anti-allergic agents, followed by H1-antihistamines [29,30,32], whereas adrenaline is used in 18–36% of patients depending on the severity of anaphylaxis and case selection, despite historical concerns regarding its potential to aggravate myocardial ischemia and coronary vasospasm [29,31,35]. Regardless of the specific regimen, patients should undergo at least 4 h of observation after anaphylaxis [193].

Cardiovascular therapies are reported in 60–75% of patients and include antiplatelet agents (55–60%), anticoagulants (18–30%), nitrates (20–30%), calcium-channel blockers (CCB) (12%), statins (15%), and standard anti-ischemic therapies [29,30,32,35,42]. Although anti-allergic therapy represents a common therapeutic cornerstone across all KS phenotypes, management of the ACS component should be individualized according to the underlying anatomical and pathophysiological subtype. Non-pharmacological interventions are used in nearly half of cases (48.2%), although PCI rates vary widely across studies, from 7.5% in some recent systematic reviews to approximately 22–31% in broader adult/mixed cohorts. Revascularization strategy is largely determined by the underlying KS subtype. While invasive treatment is uncommon in type I KS and exceptionally rare in pediatric patients, PCI is required in approximately 22–31% of adult patients, and is particularly frequent in patients with type II–III, where plaque rupture with coronary or stent thrombosis is the pathogenetic substrate [30,31,32,33,35]. CABG and thrombolysis are only occasionally (1.8%) required [31]. For chest pain management, opioids such as morphine, codeine, and meperidine should be avoided because they can worsen the allergic reaction by promoting mast-cell degranulation. Intravenous paracetamol is also not recommended, as it may cause hypotension [1].

**Figure 4:**
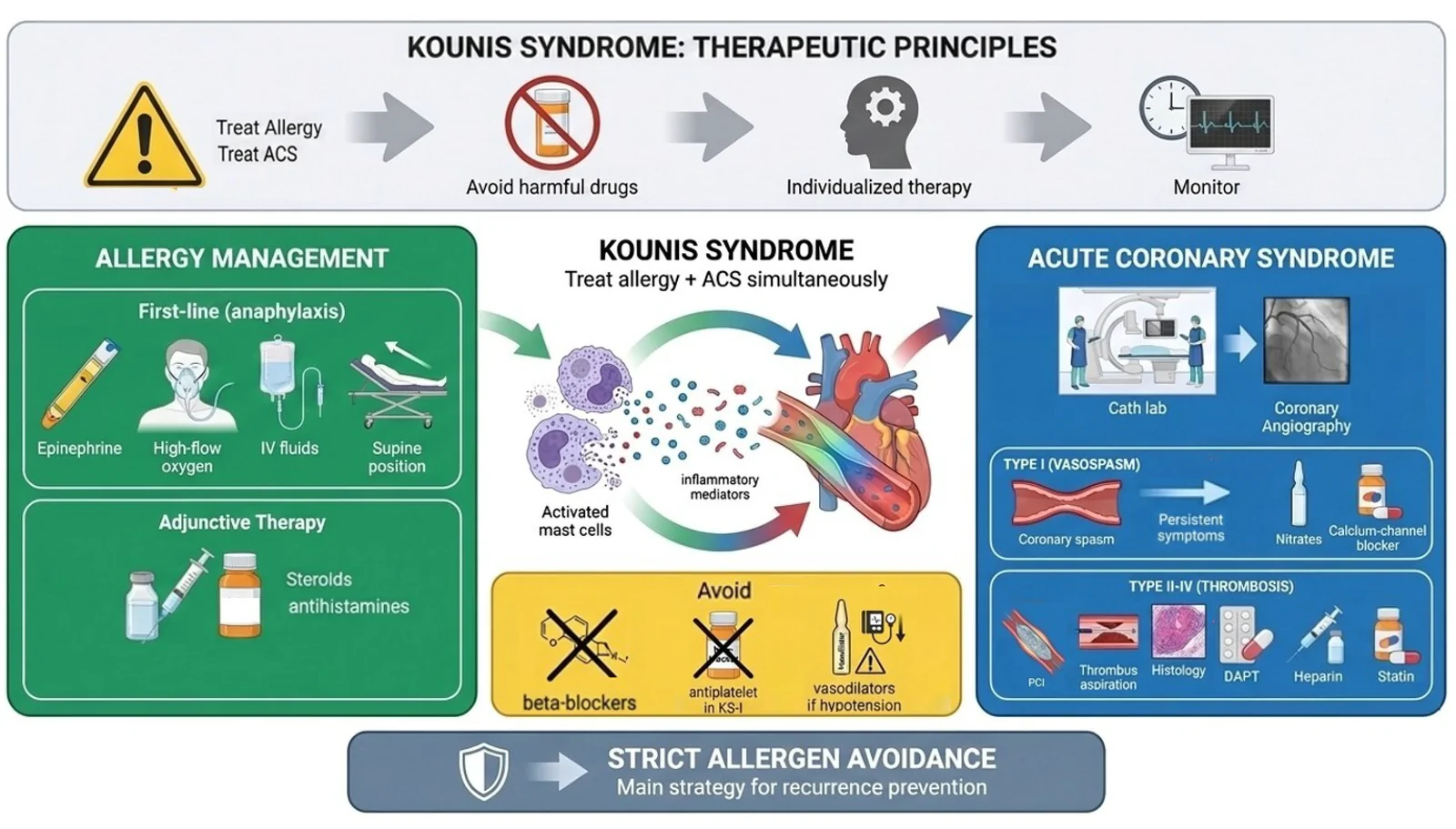
Integrated therapeutic approach to KS. The algorithm summarizes the contemporary management of KS, highlighting the simultaneous treatment of the allergic reaction and the acute coronary syndrome according to the underlying subtype. Initial management includes prompt stabilization and anti-allergic therapy, whereas coronary treatment differs between vasospastic (type I) and thrombosis-related forms (type II to IV). The figure also emphasizes therapeutic precautions, individualized treatment decisions, and strict allergen avoidance to reduce the risk of recurrence. Note: thrombus aspiration and histological examination are recommended for type III. Abbreviations: ACS, acute coronary syndrome; DAPT, dual antiplatelet therapy; KS, Kounis syndrome; PCI, percutaneous coronary intervention.

Given the dual allergic and cardiovascular nature of KS, treatment requires an integrated strategy that simultaneously addresses the hypersensitivity reaction and the ACS while accounting for the specific KS subtype as summarized in the practical management algorithm in Figure 4.

### 5.2. Allergy: Therapeutic Strategies

Anti-allergic therapy constitutes the cornerstone of management across all forms of KS and should therefore be initiated as early as possible, immediately upon recognition of the allergic reaction. However, it remains uncertain whether prompt anti-allergic treatment is capable of preventing the development of the most severe cardiovascular complications associated with the syndrome.

In type I KS, anti-allergic therapy alone may resolve symptoms and reverse electrocardiographic abnormalities, suggesting complete resolution of the underlying coronary vasospasm [194]. Intravenous corticosteroids (e.g., hydrocortisone 1–2 mg/kg/day or methylprednisolone 80–125 mg) and H_1_/H_2_ histamine receptor antagonists (e.g., diphenhydramine 1–2 mg/kg) or potent alkylamine first-generation H1 antihistamine (chlorpheniramine maleate 10–20 mg), administered intravenously slowly rather than as a bolus, or intramuscularly) are often sufficient [42,189,195]. In less severe cases, oral steroids (e.g., prednisone 0.5–1 mg/kg) may be considered [18,42]. The role of corticosteroids in KS deserves further investigation. Classic concerns related to steroid administration during acute MI (e.g., risk of myocardial rupture, delayed healing) have not been substantiated by robust meta-analytic data [192,196], and available evidence suggests that steroids are probably appropriate and safe in this context and are very commonly used [18,31,32,35].

Diagnosis of anaphylaxis or anaphylactic shock is clinical, based on combinations of cutaneous, respiratory, circulatory, and gastrointestinal symptoms (peripheral vasodilatation, hypotension, tachycardia, laryngeal oedema, bronchospasm, respiratory failure); no single sign is pathognomonic [193]. Therefore, current anaphylaxis guidelines should be followed [197]. Basic supportive measures include:

- high-flow oxygen;
- supine positioning with leg elevation (Trendelenburg);
- rapid infusion of intravenous crystalloids (normal saline) to support blood pressure and venous return.

According to contemporary recommendations, antihistamines should not delay or replace adrenaline, as they mainly provide adjunctive control of cutaneous symptoms. Their role is now regarded as secondary. Steroids are no longer recommended as routine agents for all anaphylaxis cases, because robust evidence is lacking that they prevent biphasic reactions or significantly shorten symptom duration; they should, however, be considered in refractory anaphylaxis or in patients with poorly controlled asthma [198].

Epinephrine (adrenaline) is the first-line treatment, as it increases heart rate and contractility, produces bronchodilation, and induces peripheral vasoconstriction via α_1_ receptors, thus counteracting the main pathophysiological mechanisms of anaphylaxis. It must be administered immediately once anaphylaxis is suspected, without waiting for further deterioration. In adult patients, the preferred route is intramuscular, 0.3–0.5 mg (0.3–0.5 mL of 1:1000 aqueous solution, 0.1%) injected into the anterolateral thigh. If symptoms persist or worsen, doses may be repeated every 5 min [197,198]. However, it may be ineffective or less effective in KS patients on chronic beta-blocker therapy, who may benefit from intravenous glucagon [1,18]. Epinephrine represents one of the most controversial therapies in KS. Multiple reports, together with pathophysiological and pharmacodynamic evidence and experts’ opinions, have raised concerns that adrenaline administration may exacerbate coronary vasospasm and myocardial ischemia in patients with KS [18,173,186,188,199,200]. Mechanistically, epinephrine can lead to myocardial ischemia by promoting platelet activation and aggregation (through specific receptors, thromboxane A_2_, increased sensitivity to adenosine, and enhanced fibrinogen binding). Thus, according to a traditional view, ACS occurring during an allergic reaction may be paradoxically worsened, or even provoked, by routine adrenaline use in some susceptible individuals [1,186,199,201,202,203]. However, distinguishing the natural progression of KS from the potential effects of adrenaline may be challenging, and a mere temporal association should not be considered sufficient evidence of causality [180,204,205]. The perceived negative impact of adrenaline largely reflects its pharmacodynamic profile and the temporal association with clinical worsening in selected case reports. However, nearly all patients with anaphylaxis receive adrenaline, resulting in unavoidable indication bias. As such, it is impossible to determine with certainty whether adrenaline is causative in the progression of KS or whether deterioration reflects the natural progression of a severe allergic event that would not have been prevented even with optimal therapy. Conversely, numerous cases and recent reviews document safe use of adrenaline with good outcomes, without apparent worsening of KS [50,158,180]. In the review by Ridella on antibiotic-induced KS, 23% of patients received adrenaline, and survival was excellent [204]. As highlighted, across published series, it was administered in approximately 18–36% of unselected patients, reflecting substantial variability in clinical presentation and physician decision-making [30,31,35,42]. In contrast, selected pooled analyses enriched for drug- or contrast-induced reactions have reported markedly higher utilization rates, reaching approximately 92% among cases with available treatment data [32].

This striking variability likely reflects differences in case selection, severity of the allergic reaction, and incomplete treatment reporting rather than true differences in therapeutic practice. Epinephrine use appears to correlate with the severity of the allergic presentation, being more frequent in patients presenting with anaphylaxis, anaphylactic shock, or profound hypotension, whereas lower rates are observed in mixed populations with milder allergic manifestations or predominant cardiac presentations. This cautious use is probably related to concerns that this drug may aggravate coronary vasospasm, increase myocardial oxygen consumption, enhance platelet activation, prolong the QT interval, and promote arrhythmias. Nevertheless, despite these theoretical and observational concerns, it remains the cornerstone of treatment for life-threatening anaphylaxis, and current evidence does not support withholding its administration when severe allergic reactions are present. Therefore, management should balance the immediate risk of anaphylactic collapse against the potential for worsening coronary dysfunction, with treatment individualized according to the patient’s hemodynamic status and clinical presentation [33,80,180,199].

The same principles of anti-allergic management apply to type II to IV KS. Accordingly, prompt administration of antihistamines and corticosteroids should be considered in all patients, while intramuscular epinephrine remains indicated in selected cases presenting with severe anaphylaxis or anaphylactic shock [118,206]. However, unlike type I KS, treatment of these subtypes also requires management of the underlying coronary pathology, including antithrombotic therapy, percutaneous coronary intervention, or specific treatment of stent thrombosis when appropriate [46].

### 5.3. Specific Therapy of Coronary Vasospasm

As frequently demonstrated by negative coronary angiography performed during the acute phase, coronary vasospasm in type I KS often resolves spontaneously or following anti-allergic therapy, without the need for specific coronary vasodilator treatment.

In stable patients, often prior to angiography, many clinicians empirically administer intravenous coronary vasodilators (nitrates and/or CCB drugs verapamil or diltiazem), as in other forms of vasospastic angina. Systemic administration of these drugs must be used cautiously, and sometimes avoided, in the presence of severe hypotension, which is common in anaphylaxis [32]. Sometimes sublingual nitrates have been used with benefit in selected cases, while oral CCBs are not generally used in the acute phase [189,207]. CCBs are used in 12% of patients [33]. Intravenous nitrates should be started at low doses (5–10 μg/min) and titrated in increments of 10 μg/min every 5 min until symptom relief or onset of adverse effects [188]. When coronary spasm is directly documented angiographically, it can be treated more frequently with intracoronary nitrates [77,78] or, less frequently, with intracoronary CCB [97,208], generally with rapid and complete resolution.

As myocardial ischemia persists despite anti-allergic treatment, urgent bailout balloon angioplasty is required to restore coronary flow, as needed [209].

In refractory or recurrent allergic coronary vasospasm, which may respond poorly to conventional vasodilators, systemic corticosteroids can play an important adjunctive role, particularly in the presence of eosinophilia, leading to effective resolution of recurrent ischemic episodes [210,211,212].

After resolution of the acute episode, there is no evidence that chronic therapy with CCB or nitrates prevents further KS episodes. Such treatments are sometimes prescribed empirically by extrapolating from vasospastic angina, but data are lacking. Recurrent KS episodes are typically triggered by re-exposure to the same allergen and, therefore, do not necessarily justify chronic pharmacological prevention [50,144,207,213,214,215]. Therefore, in most type I KS patients, chronic prophylaxis is probably not useful, and strict avoidance of the allergenic trigger, almost always identifiable, remains the most effective therapy [50,144].

According to the traditional view, beta-blockers should generally be avoided in type I because they may worsen vasospasm by leaving α-adrenergic vasoconstriction unopposed [1,18,188,189,216]. Nevertheless, their use is not uncommon in clinical practice, being reported in 21.4% of KS cases, with no clear evidence linking their administration to increased mortality or adverse clinical outcomes [42]. Since type I KS is not an atherothrombotic event, treatment does not routinely require antiplatelet or anticoagulant therapy. Nevertheless, empiric intravenous aspirin and low-molecular-weight heparin or unfractionated heparin according to STEMI protocols may be administered before, or in the absence of, angiography [1]. Some clinicians have also used DAPT loading (ASA + P2Y12 inhibitor) prior to angiography, but this practice is no longer supported by the 2023 ESC ACS guidelines and should be avoided [100]. Statin and antihypertensive therapy should be tailored to global cardiovascular risk rather than KS per se. Accordingly, once obstructive coronary artery disease and plaque disruption have been excluded, the general cardiovascular management of type I should be aligned with contemporary MINOCA recommendations, with specific treatment tailored to the underlying vasomotor mechanism and to the severity of the allergic reaction [168,175,176]. In patients with severe left ventricular dysfunction, diuretics, inotropes, vasopressors, and mechanical support (e.g., intra-aortic balloon pump) should be used according to standard HF and shock recommendations. In the most severe cases complicated by cardiogenic shock or refractory cardiac arrest, advanced mechanical circulatory support may be required, including intra-aortic balloon pump (IABP), veno-arterial extracorporeal membrane oxygenation (VA-ECMO), as a bridge to recovery while controlling both the allergic reaction and the underlying coronary event [78,217].

## 6. Conclusions

Kounis syndrome is no longer a rare clinical “curiosity” but a true, though underrecognized, cause of ACS requiring greater awareness among cardiologists, internists, emergency physicians, allergists, and intensivists. Although all subtypes deserve recognition, current evidence is strongest for type I, which emerges as a distinct clinicopathological entity characterized by hypersensitivity-mediated coronary vasomotor dysfunction involving both the epicardial and coronary microvascular circulation.

The available evidence supports the type I KS variant as a specific allergic vasospastic MINOCA endotype, rather than an uncommon trigger of coronary spasm. Recognition of patients with this phenotype has important practical implications, starting from diagnosis to the decision for urgent interventional treatment in view of ruling out obstructive coronary artery disease. A modern and structured diagnostic workup is necessary, integrating an in-depth recognition of anaphylaxis, clinical manifestations, standard diagnostics, cardiac biomarkers, advanced imaging, and—whenever appropriate—coronary functional testing. Based on the present review, allergy-mediated coronary syndromes should be carefully considered in future ACS classifications for endorsement into the INOCA/MINOCA setting with more specific diagnostic and therapeutic algorithms. Prospective registries and mechanistic research are encouraged to improve patient outcomes.

## Figures and Tables

**Table 1:** Major systematic reviews, pooled analyses, and clinical cohorts reporting the epidemiology and outcomes of KS. Data are presented as reported by the original authors. Variations in subtype distribution should be interpreted in light of differences in study design, inclusion criteria, and diagnostic definitions. * Quetsch et al. [36] included exclusively patients with allergic vasospastic angina (type I); therefore, type II KS and type III KS cases were excluded by design. † Dogan et al. [42] represents a retrospective consecutive real-world cohort of patients with KS and not a systematic review of published cases. Abbreviation: KS, Kounis syndrome.

First Author	Year	Period of Analysis	Total Cases	Type I n (%)	Type II n (%)	Type III n (%)	Unclassified n (%)	Male– Female %	Mortality Rate (%)	Mortality by Subtype
Abdelghany [29]	2017	≤March 2016	175	127 (72.6)	39 (22.3)	9 (5.1)	—	74.3–25.7	2.9	—
Roumeliotis [30]	2021	≤January 2020	288	166 (57.6)	71 (24.7)	19 (6.6)	32 (11.1)	71.2–28.8	—	—
Youcefi [31]	2024	≤September 2023	350	255 (72.9)	67 (19.1)	28 (8.0)	—	—	4.8	—
Yakushin [32]	2024	≤November 2023	235	117 (49.7)	64 (27.2)	14 (5.9)	38 (16.2)	68.5–31.5	5.5	—
Quetsch * [36]	2025	1992–2022	393	393 (100)	—	—	—	64–36	—	—
Cahuapaza-Gutierrez [35]	2025	≤March 2024	214	93 (43.5)	51 (23.8)	19 (8.9)	51 (23.8)	69.6–30.4	7.5	—
Rochel- Perez [33]	2025	2018–2023	155	71 (46.1)	41 (26.4)	22 (14.1)	21 (13.5)	66.6–33.4	—	I: 1.4%; II: 0%; III: 18%
Dogan † [42]	2025	2018–2024	28	25 (89.3)	3 (10.7)	0	—	64.3–35.7	0	No deaths

**Table 2:** Reported triggers of KS. The table summarizes the broad spectrum of triggers reported in KS. Although drugs remain the most frequently implicated category, a wide range of non-pharmacological triggers, including insect stings, foods, contrast media, environmental allergens, and coronary biomaterials, have also been associated with the syndrome. This diversity supports the concept that KS represents a final common pathway of mast-cell activation and allergic inflammation capable of precipitating coronary vasospasm, plaque destabilization, thrombosis, or stent-related events. Abbreviations: ACE, angiotensin-converting enzyme; COVID-19, coronavirus disease 2019; KS, Kounis syndrome; NSAIDs, non-steroidal anti-inflammatory drugs.

Category	Representative Triggers
Antibiotics	Penicillin, ampicillin, amoxicillin, ampicillin/sulbactam, piperacillin/tazobactam, sulbactam/cefoperazone, cefazolin, cefoxitin, cefuroxime, cefradine, ceftriaxone, amikacin, clindamycin, lincomycin, clarithromycin, telithromycin, ciprofloxacin, metronidazole, trimethoprim–sulfamethoxazole, vancomycin
Antiviral agents	Oseltamivir, brivudine
Antifungal agents	Fluconazole, amphotericin B
Non-steroidal anti-inflammatory drugs (NSAIDs) and analgesics	Aspirin, ibuprofen, diclofenac, ketorolac, naproxen, metamizole, paracetamol/acetaminophen
Cardiovascular medications	ACE inhibitors, β-blockers, calcium-channel blockers
Antiplatelet and antithrombotic agents	Aspirin, clopidogrel, prasugrel, ticagrelor, low-molecular-weight heparins
Anaesthetic and peri-procedural drugs	Propofol, etomidate, midazolam, fentanyl, rocuronium, succinylcholine, lidocaine, bupivacaine
Antineoplastic and biological agents	Cisplatin, oxaliplatin, carboplatin, cyclophosphamide, 5-fluorouracil, paclitaxel, rituximab, trastuzumab, infliximab, epirubicin
Gastrointestinal medications	Lansoprazole and other proton-pump inhibitors, ranitidine
Other medications	Tramadol, glucocorticoids, oral contraceptives, adrenaline (epinephrine), allopurinol
Vaccines	Influenza vaccine, COVID-19 vaccines, tetanus-containing vaccines, pneumococcal vaccine
Intravenous contrast media	Iodinated contrast agents used during coronary angiography, computed tomography and other radiological procedures; ultrasound contrast agents used during echocardiography
Skin disinfectants and antiseptics	Chlorhexidine, povidone–iodine
Coronary devices and biomaterials	Drug-eluting stents, bare-metal stents, metallic stent components (nickel, chromium, cobalt)
Animal stings and bites	Bees, wasps, other Hymenoptera species, scorpions, snakes, black widow spiders, jellyfish
Foods	Shellfish, shrimp, crab, lobster, fish, mushrooms, kiwi, peach, apple, grape, vegetables, peanuts, tree nuts, chocolate, milk products
Environmental allergens	Latex, pollen, house dust mites, animal dander
Occupational and chemical exposures	Hair dyes, industrial chemicals, cleaning agents, insecticides, pesticides
Systemic allergic disorders and mast-cell activation syndromes	Anaphylaxis of any cause, mastocytosis, mast-cell activation syndromes, idiopathic allergic reactions

## Data Availability

No new data were created or analyzed in this study. Data sharing is not applicable to this article.

## Supplementary Materials

The following supporting information can be downloaded at: https://www.mdpi.com/article/10.3390/jcm15166417/s1, Supplementary Table S1. Principal biochemical mediators involved in the allergic cascade of KS. Mast cell activation represents the central pathophysiological event in KS. The release of preformed mediators (e.g., histamine, tryptase, chymase), newly synthesized lipid mediators (e.g., platelet-activating factor, leukotrienes, prostaglandins), cytokines, chemokines, hematopoietic factors, and growth factors promotes coronary vasoconstriction, endothelial dysfunction, inflammatory cell recruitment, platelet activation, plaque destabilization, and thrombus formation. These mechanisms contribute to the development of coronary vasospasm in type I, plaque erosion or rupture in type II, and stent thrombosis or restenosis in type III. Abbreviations: bFGF, basic fibroblast growth factor; CCL, CC chemokine ligand; CXCL, CXC chemokine ligand; GM-CSF, granulocyte–macrophage colony-stimulating factor; KS, Kounis syndrome; MCP-1, monocyte chemoattractant protein-1; MMP, matrix metalloproteinase; PAF, platelet-activating factor; PDGF, platelet-derived growth factor; TNF-α, tumour necrosis factor-α; VEGF, vascular endothelial growth factor.

## Abbreviations

The following abbreviations are used in this manuscript:

ACC	American College of Cardiology
ACS	Acute Coronary Syndrome
Ach	Acetylcholine
AHA	American Heart Association
ANOCA	Angina with Non-Obstructive Coronary Arteries
BMIPP	β-Methyl-p-Iodophenyl-Pentadecanoic Acid
CABG	Coronary Artery Bypass Grafting
CAD	Coronary Artery Disease
CCB	Calcium Channel Blocker
CCTA	Coronary Computed Tomography Angiography
CFR	Coronary Flow Reserve
CK-MB	Creatine Kinase MB Isoenzyme
CMD	Coronary Microvascular Dysfunction
CMR	Cardiac Magnetic Resonance
COVADIS	COronary VAsomotion Disorders International Study Group
CT	Computed Tomography
DES	Drug-Eluting Stent
ECG	Electrocardiogram
ESC	European Society of Cardiology
FFR	Fractional Flow Reserve
hs-Tn	High-Sensitivity Cardiac Troponin
IgE	Immunoglobulin E
IL	Interleukin
IMR	Index of Microcirculatory Resistance
INOCA	Ischemia with Non-Obstructive Coronary Arteries
IVUS	Intravascular Ultrasound
KS	Kounis Syndrome
LAD	Left Anterior Descending (coronary artery)
LGE	Late Gadolinium Enhancement
LVEF	Left Ventricular Ejection Fraction
MI	Myocardial Infarction
MINOCA	Myocardial Infarction with Non-Obstructive Coronary Arteries
MRGPRX2	Mas-Related G Protein-Coupled Receptor Member X2
NSAIDs	Non-Steroidal Anti-Inflammatory Drugs
OCT	Optical Coherence Tomography
PAF	Platelet-Activating Factor
PEA	Pulseless Electrical Activity
PCI	Percutaneous Coronary Intervention
RCA	Right Coronary Artery
SPECT	Single-Photon Emission Computed Tomography
STEMI	ST-Segment Elevation Myocardial Infarction
TNF-α	Tumor Necrosis Factor Alpha
