# Supplementary material for: Kounis Syndrome in the Modern Era: A Comprehensive Review of Allergic Acute Coronary Syndromes
Source: J Clin Med. 2026 Aug 19;15(16):6417. doi: 10.3390/jcm15166417 (PMC13513222; doi:10.3390/jcm15166417)
Supplement: Supplementary file 1 [file jcm-15-06417-s001.zip › jcm-4483632-supplementary.pdf]

Table S1. Principal biochemical mediators involved in the allergic cascade of KS. Mast cell activation represents the central pathophysiological event in KS. The release of preformed mediators (e.g., histamine, tryptase, chymase), newly synthesized lipid mediators (e.g., platelet-activating factor, leukotrienes, prostaglandins), cytokines, chemokines, hematopoietic factors, and growth factors promotes coronary vasoconstriction, endothelial dysfunction, inflammatory cell recruitment, platelet activation, plaque destabilization, and thrombus formation. These mechanisms contribute to the development of coronary vasospasm in type I, plaque erosion or rupture in type II, and stent thrombosis or restenosis in type III. Abbreviations: bFGF, basic fibroblast growth factor; CCL, CC chemokine ligand; CXCL, CXC chemokine ligand; GM-CSF, granulocyte-macrophage colony-stimulating factor; KS, Kounis syndrome; MCP-1, monocyte chemoattractant protein-1; MMP, matrix metalloproteinase; PAF, platelet-activating factor; PDGF, platelet-derived growth factor; TNF-α, tumour necrosis factor-α; VEGF, vascular endothelial growth factor.

Category	Mediator	Main Cellular Source	Cardiovascular Effects	Potential Role in Kounis Syndrome
Preformed mediator	Histamine	Mast cells, basophils	Coronary vasoconstriction (H1-mediated), endothelial dysfunction, platelet activation, increased vascular permeability	Major trigger of coronary vasospasm and myocardial ischaemia
Preformed mediator	Tryptase	Mast cells	Activation of matrix metalloproteinases (MMPs), extracellular matrix degradation	Plaque destabilization, erosion, and rupture, particularly in type II and III KS
Preformed mediator	Chymase	Mast cells	Angiotensin II generation, vasoconstriction, endothelial dysfunction	Coronary spasm and plaque instability
Preformed mediator	Cathepsin G	Mast cells, neutrophils	Platelet activation and thrombin generation	Promotion of coronary thrombosis
Preformed mediator	Carboxypeptidase	Mast cells	Regulation of vasoactive peptides and inflammatory pathways	Amplification of allergic vascular injury
Proteoglycan	Heparin	Mast cells	Interaction with coagulation and inflammatory cascades	Modulation of thrombo-inflammatory responses
Proteoglycan	Chondroitin sulfate	Mast cells	Extracellular matrix remodelling	Vascular inflammation and tissue remodelling
Lipid mediator	Platelet-activating factor (PAF)	Mast cells, basophils, macrophages, platelets	Potent platelet aggregation, vasoconstriction, increased permeability	Central mediator linking anaphylaxis with coronary thrombosis

Category	Mediator	Main Cellular Source	Cardiovascular Effects	Potential Role in Kounis Syndrome
Lipid mediator	Leukotriene C4 (LTC4)	Mast cells, eosinophils	Intense coronary vasoconstriction	Coronary vasospasm and myocardial ischaemia
Lipid mediator	Leukotriene B4 (LTB4)	Mast cells, eosinophils, neutrophils	Leukocyte recruitment and activation	Amplification of vascular inflammation
Lipid mediator	Prostaglandin D2 (PGD2)	Mast cells	Alteration of coronary vascular tone and inflammatory cell recruitment	Coronary vasomotor dysfunction
Lipid mediator	Prostaglandin E2 (PGE2)	Mast cells, macrophages	Modulation of vascular tone and inflammation	Microvascular dysfunction
Lipid mediator	Thromboxane A2	Activated platelets	Potent vasoconstriction and platelet aggregation	Enhancement of coronary thrombosis
Cytokine	TNF-α	Mast cells, macrophages, T lymphocytes	Endothelial activation, inflammatory amplification, procoagulant state	Sustains vascular inflammation and plaque vulnerability
Cytokine	IL-4	Th2 lymphocytes, mast cells	Promotion of IgE-mediated immune responses	Maintenance of allergic inflammation
Cytokine	IL-5	Th2 lymphocytes, eosinophils	Eosinophil recruitment and activation	Eosinophilic vascular injury
Cytokine	IL-6	Mast cells, macrophages	Acute-phase inflammatory response	Plaque destabilization and vascular inflammation
Cytokine	IL-13	Th2 lymphocytes	Allergic inflammation and vascular remodelling	Sustained allergic response
Chemokine	MCP-1 (CCL2)	Endothelial cells, macrophages	Monocyte recruitment	Vascular inflammation and plaque activation
Chemokine	IL-8 (CXCL8)	Endothelial cells, macrophages	Neutrophil chemotaxis and activation	Amplification of coronary inflammation
Chemokine	RANTES (CCL5)	T lymphocytes, platelets	Leukocyte and platelet recruitment	Thrombo-inflammatory response
Hematopoietic factor	IL-3	T lymphocytes, mast cells	Expansion of mast cells and basophils	Sustained allergic activation
Hematopoietic factor	GM-CSF	T lymphocytes, macrophages	Leukocyte activation and survival	Persistence of inflammatory response
Growth factor	VEGF	Mast cells, macrophages	Increased vascular permeability and angiogenesis	Vascular leakage and oedema
Growth factor	TGF-β	Mast cells, macrophages	Fibrosis and vascular remodelling	Chronic vascular changes

Category	Mediator	Main Cellular Source	Cardiovascular Effects	Potential Role in Kounis Syndrome
Growth factor	PDGF	Platelets, macrophages	Smooth muscle proliferation	Vascular remodelling and restenosis
Growth factor	bFGF	Mast cells, endothelial cells	Angiogenesis and smooth muscle proliferation	Vascular remodelling
